# Discovery of a Small Molecule with an Inhibitory Role for RAB11

**DOI:** 10.3390/ijms252313224

**Published:** 2024-12-09

**Authors:** Camille Lempicki, Julian Milosavljevic, Christian Laggner, Simone Tealdi, Charlotte Meyer, Gerd Walz, Konrad Lang, Carlo Cosimo Campa, Tobias Hermle

**Affiliations:** 1 Renal Division, Department of Medicine, Faculty of Medicine, Medical Center, University of Freiburg, Hugstetter Strasse 55, 79106 Freiburg, Germany; camille.lempicki@uniklinik-freiburg.de (C.L.); julian.milosavljevic@uniklinik-freiburg.de (J.M.);; 2 Atomwise Inc., 250 Sutter St #650, San Francisco, CA 94108, USA; 3 Italian Institute for Genomic Medicine, Str. Prov. le 142, km 3.95, 10060 Candiolo, Turin, Italycarlocosimo.campa@iigm.it (C.C.C.); 4 Department of Mechanical and Aerospace Engineering, Politecnico di Torino, Corso Duca degli Abruzzi 24, 10129 Turin, Turin, Italy; 5 CIBSS—Centre for Integrative Biological Signalling Studies, 79104 Freiburg, Germany; 6 Candiolo Cancer Institute, FPO-IRCCS, 10060 Candiolo, Turin, Italy

**Keywords:** Rab11, endocytosis, drug discovery, high-throughput screening, virtual screening, machine learning, *Drosophila* nephrocytes

## Abstract

RAB11, a pivotal RabGTPase, regulates essential cellular processes such as endocytic recycling, exocytosis, and autophagy. The protein was implicated in various human diseases, including cancer, neurodegenerative disorders, viral infections, and podocytopathies. However, a small-molecular inhibitor is lacking. The complexity and workload associated with potential assays make conducting large-scale screening for RAB11 challenging. We employed a tiered approach for drug discovery, utilizing deep learning-based computational screening to preselect compounds targeting a specific pocket of RAB11 protein with experimental validation by an in vitro platform reflecting RAB11 activity through the exocytosis of GFP. Further validation included the exposure of *Drosophila* by drug feeding. In silico pre-screening identified 94 candidates, of which 9 were confirmed using our in vitro platform for Rab11 activity. Focusing on compounds with high potency, we assessed autophagy, which independently requires RAB11, and validated three of these compounds. We further analyzed the dose–response relationship, observing a biphasic, potentially hormetic effect. Two candidate compounds specifically caused a shift in Rab11 vesicles to the cell periphery, without significant impact on Rab5 or Rab7. *Drosophila* larvae exposed to another candidate compound with predicted oral bioavailability exhibited minimal toxicity, subcellular dispersal of endogenous Rab11, and a decrease in RAB11-dependent nephrocyte function, further supporting an inhibitory role. Taken together, the combination of computational screening and experimental validation allowed the identification of small molecules that modify the function of Rab11. This discovery may further open avenues for treating RAB11-associated disorders.

## 1. Introduction

Endocytosis is initiated by the invagination of small patches of the cell membrane to form small transport vesicles. These are processed by a complex molecular machinery for transfer and sorting either towards degradation in the lysosome or recycling back to the plasma membrane [1]. This cellular logistics network is essentially orchestrated by the Rab proteins [2]. Over 60 members of this family of small GTPases act as molecular switches that define the identity of the transport vesicle and/or the destination membrane. Rab proteins interact with a wide range of effectors to exercise their function [3]. Rab GTPase family members RAB11A and RAB11B share ~89% of their sequence in humans. Both proteins contribute significantly to a variety of cellular functions, particularly endocytic recycling, exocytosis, and autophagy [4,5,6,7,8,9]. Endocytosis is crucial for the proper function of the glomerular filtration barrier [10,11,12]. We recently identified variants in the RAB11 inhibitor gene *TBC1D8B* as the cause of a hereditary nephrotic syndrome [13,14,15]. This sparked our interest in modulating RAB11 activity but to the best of our knowledge, specific small-molecule inhibitors were unavailable. Assessment of RAB11 function is challenging, making large-scale drug discovery difficult. Considering the suggested role of RAB11 in a range of human diseases, including cancer [16], neurodegenerative diseases [17], and infections [18], a small-molecule inhibitor would be highly desirable and might potentially be of therapeutic value. Recent advances suggest that a combined approach employing computational drug screening may offer a potential solution to this challenge [19].

Here, we used deep learning-based computational drug screening to identify a set of candidates. For validation, we applied a newly established screening platform for endogenous Rab11 based on exocytosis of a secretory variant of GFP in human embryonic kidney cells (HEK293T). We identified nine hits and selected compounds for higher potency. We validated three compounds studying basal autophagy as a second, independent cellular function that requires RAB11. Subcellular localization of Rab11 was further specifically affected in cells or *Drosophila* nephrocytes. We explored the dose–response relationship of three of these candidates and identified a biphasic, possibly hormetic response. Being commercially available, the novel inhibitors can serve as immediately useful tools for research purposes.

## 2. Results

### 2.1. Establishment of a Screening Platform for RAB11 and Computational Screening

**Figure 1:**
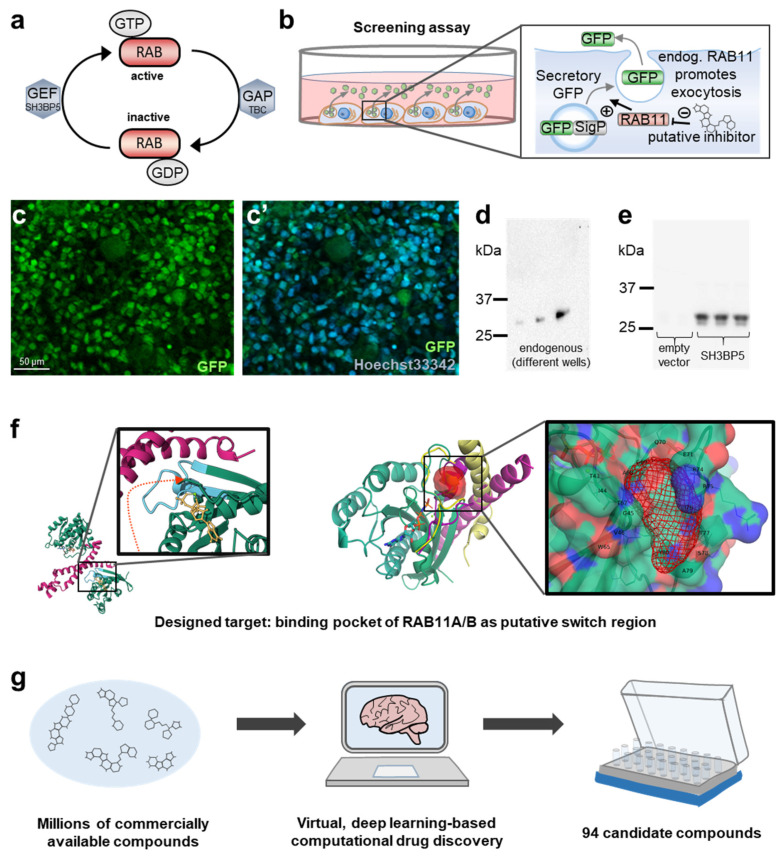
A screening platform for RAB11 and computational drug screening. (**a**) Schematic shows cycling of RAB proteins between the active state (GTP-bound) that is terminated by support of GAPs by hydrolysis of GTP to GDP, which in turn is displaced by GEFs to allow reactivation by GTP binding. (**b**) Schematic illustrates the screening platform. HEK293T cells express a secretory GFP that carries an *IFNA2*-derived signal peptide. Secretion is promoted by RAB11, so that reduction in GFP secretion into the supernatant indicates reduced activity of RAB11. (**c**,**c’**) Fluorescence microscopy image of HEK293T cells stably transduced with the secretory GFP shows that all cells are GFP-positive by comparing the nuclear stain (blue) with the green channel. (**d**) Immunoblotting with anti-GFP using cellular supernatants from different wells with HEK293T cells stably transduced with secretory GFP reveals strong, random variation in GFP positivity between different wells. (**e**) Transient transfection of HEK293T cells from (**c**,**d**) with RAB11-GEF SH3BP5 or empty vector shows strong increase in GFP from the cellular supernatant with activation of RAB11 after immunoblotting. (**f**) This panel illustrates the target domain on RAB11 used for computational screening. On the left, the structure of RAB11A is shown as a dimer (green) in complex with the effector FIP3 (PBD ID: 2HV8). The enlargement illustrates the binding pocket near GTP (yellow) and two switch regions. The targeted residues are blue. The right side of the panel shows the structure of RAB11B (green, PBD ID: 2F9M) with the binding pocket highlighted in red and overlay of RAB11A/FIP3 complex in yellow (PDB ID 2HV8, active form binding GTP) and the RAB11B/PKG II in magenta (PDB ID 4OJK, inactive form binding GDP) to illustrate the flexibility of the switch region and its interaction with effector proteins. The enlargement illustrates the binding pocket with the targeted residues. (**g**) The schematic illustrates the deep learning-based computational high-throughput screening using the AtomNet^®^ technology (Atomwise Inc., San Francisco, CA, USA). Millions of commercially available compounds are screened virtually against the target structure before the selection of 94 compounds that are purchased for further testing.

Rab proteins shuttle between the active state binding GTP and the inactive state binding GDP. Shuttling is regulated by activating guanine nucleotide exchange factors (GEFs) and inhibitory GTPase-activating proteins (GAPs, Figure 1a). Recruitment of effectors mediates downstream effects. We previously identified TBC1D8B as a regulator of RAB11A and RAB11B, and disinhibition of RAB11 by DNA variants in *TBC1D8B* entailed kidney dysfunction [13,14]. Since RAB11 further associates with other diseases [16,17,18], our goal was to identify a small-molecular inhibitor of RAB11. However, screening for RAB11 activity is nontrivial. Localization of RAB11 provides only imprecise information concerning its activation state and radioactivity assays in cell-free environments are impractical and necessitate protein purification [20,21,22]. Using pull-down experiments with effectors such as FIP3 might compete with the optimal binding sites of an inhibitor in an overexpression setting [22]. Thus, indirect measurements are the more suitable strategy. To obtain a functional assay for RAB11, we previously introduced the signal peptide of interferon alpha-2 (*IFNA2*) into the N-terminus of emerald GFP [13] (secretory GFP), which can be used as a readout for RAB11 activity (Figure 1b). The appearance of GFP in the supernatant following exocytosis reflects the activity of RAB11, since exocytosis depends on RAB11. Exocyst-related assays have previously been proposed to study the function of RAB11 [21]. A decrease in secretory GFP in the cellular supernatant suggests efficient inhibition of RAB11 (Figure 1b).

Transient transfection of secretory GFP yielded variable results, rendering it unsuitable for screening purposes. Thus, we used lentiviral transduction of HEK293T cells (HEK cells) and selected one highly GFP-expressing clone (Supplementary Figure S1a). Despite efficient and complete transduction of the resultant HEK cell line (Figure 1c,c’), the GFP protein levels in the cell culture supernatant were highly variable under basal, unstimulated conditions (Figure 1d). To overcome this impediment, we transiently transfected the stable cells with SH3 Domain Binding Protein 5 (*SH3BP5*), an established RAB11-GEF. Expression of the GEF protein, which promotes the active state of the endogenous RAB11, resulted in consistently elevated amounts of secretory GFP in the supernatant of the stable cell line (Figure 1e). We compared positive and negative controls (*SH3BP5* vs. empty vector) and determined a Z-factor of 0.605 for this assay (Supplementary Figure S1b). This suggests a discriminative test useful for distinguishing between effective and ineffective compounds. Having established a platform that is suitable for low-throughput screening, we employed the proprietary AtomNet [23] artificial intelligence platform that utilizes a convolutional neural network. This allows the computational screening of millions of compounds for targeted protein domains before purchasing only select compounds for in vitro screening. This approach has previously been applied successfully [24,25,26]. The AtomNet model had originally been trained against several million small molecules and protein structures, enabling it to predict compounds effective against unfamiliar targets. The screen was designed based on the crystallized structure of active human RAB11B [27] (protein data bank [PBD] ID: 2F9M). In this active, GTP-bound conformation, two switch regions form a small binding site that appears targetable with small molecules. Thus, the area selected for virtual screening is defined by the residues T43, I44, G45, V46, W65, T67, A68, G69, Q70, E71, R74, R/A75, I76, T77, S78, A79, and Y80 that are situated in a pocket between two promising switch regions [28] compared with the inactive form of RAB11B [27] (PBD ID: 2F9L, Figure 1f). The respective domain was further aligned to the RAB11A/FIP3 (PBD ID: 2HV8) [29] and RAB11B/PKG II (PBD ID: 4OJK) [30] complexes to illustrate the flexibility of the switch region and its interaction with effector proteins. In order to form the effector complexes, the flexible protein loops have to undergo significant structural changes. Locking the switch region with a small molecule may prevent effector recruitment and thus provide an effective way to block the function of RAB11 (Figure 1f). The sequence of the pocket region is conserved between both variants of RAB11 (Supplementary Figure S1c). Since both proteins are nearly identical in the targeted region, an effect on both paralogs can be expected, which is useful to avoid functional compensation. The virtual screen with the AtomNet model identified 94 commercially available chemical compounds predicted to bind at the intended site on RAB11B, which were purchased for testing (Figure 1g, Supplementary Table S1).

### 2.2. In Vitro Candidate Screen

**Figure 2:**
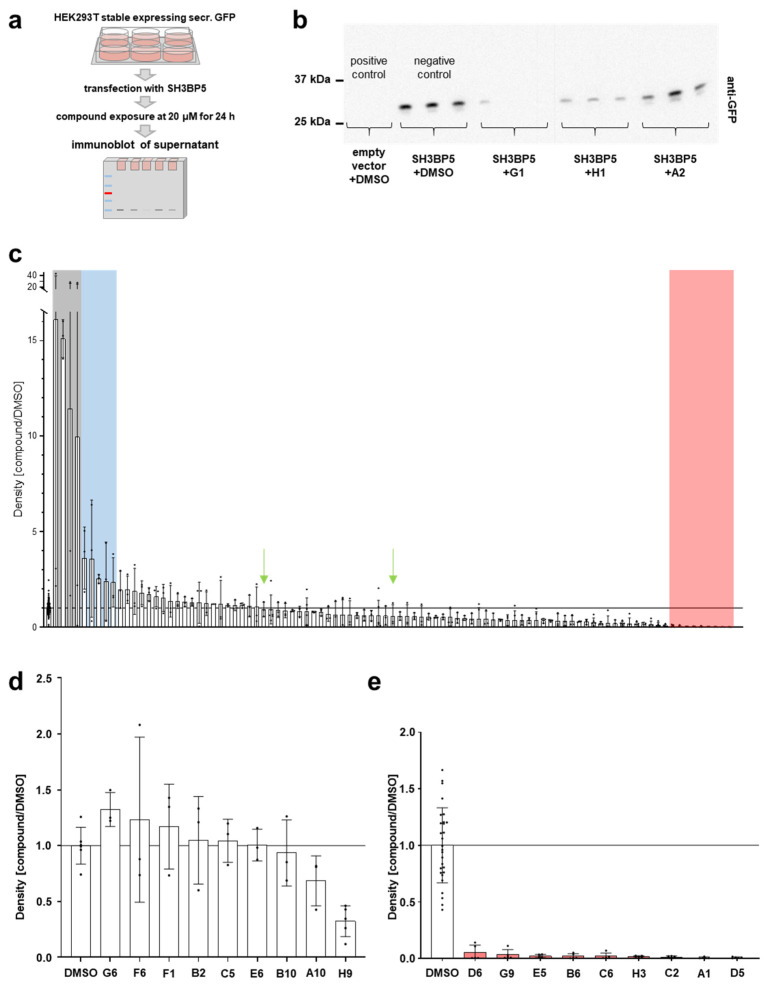
Screening identifies nine hits as inhibiting compounds. (**a**) Schematic illustrates the work flow of in vitro screening of compounds. (**b**) Representative Western blot shows GFP bands in the supernatant as an example. GFP is undetectable in control conditions (empty vector) but strongly enhanced after activation of RAB11 by transfection of GFP-*SH3BP5* with DMSO (vehicle), while compounds reduce secretion of GFP to a variable extent. (**c**) Quantification of immunoblot for all tested compounds analogous to (**b**) showing repeat measurements of compounds after censoring four compounds with excessively overshooting GFP secretion. Statistical significance was defined as *p* < 0.01; individual *p*-values, see Supplementary Table S2. Bars marked in green represent the blinded negative controls. Grey background indicates control, blue background significant increase, red background significant decrease in GFP secretion. Censored: F6, A10, G6, and B10. Significantly activating, from left to right: B2, H9, C5, E6, and F1. Not significant, from left to right: F9, E1, E10, D12, C7, E7, B12, H2, B5, E9, A4, D2, C4, B4, D4, G11, A11, E12, A3, H6, H12 (blinded control 1), D7, B9, F10, A8, A12, G8, G10, A7, E11, F5, F3, G5, E4, B1, D10, D8, F8, G12 (blinded control 2), A2, B8, D11, H10, F11, F4, C9, H8, G4, D9, H7, D3, E2, H5, C11, B3, G3, A6, A9, E8, A5, D1, C12, F7, C8, H4, G7, H11, F12, B7, E3, F2, C3, G2, H1, G1, B11, and C10. Significantly inhibiting, from left to right: D6, G9, E5, B6, C6, H3, C2, D5, and A1. (**d**) Quantification of immunoblots analogous to (**b**) of nine compounds with an initially overshooting response show normal or even reduced activity after replicate measurement that did not differ significantly from the control (DMSO, mean ± SD, *n* = 3–5 per condition, *p* > 0.05 for all compounds). This suggests unspecific toxicity as the cause of the previously observed excessive response. (**e**) Quantification of immunoblots analogous to (**b**) shows selection of nine hits with significant reduction in GFP secretion after censoring overshooting responses (mean ± SD). Statistical significance was defined as *p* < 0.01; individual *p*-values, see Supplementary Table S2.

After virtual preselection, two DMSO-only samples were added as blinded negative controls, bringing the total library to 96 compounds. We transfected HEK293T cells stably expressing secretory GFP with SH3BP5 and exposed them to the respective compounds at 20 µM for 24 h. Each immunoblot used to detect GFP secretion in the cellular supernatant included an empty vector as a positive control (low endogenous RAB11 without SH3BP5, indicating inhibition) and DMSO (vehicle) as a negative control (no reduction in SH3BP5-induced activity, work flow shown in Figure 2a). Each compound was tested in triplicates and the density of the GFP band was normalized for each compound to the respective result with DMSO alone (representative membrane Figure 2b). The screen was not designed for the identification of activators of RAB11, but surprisingly, we observed an excessive increase in GFP secretion compared to the negative control for four compounds. This made an assessment of the dataset after quantification in its entirety difficult (Supplementary Figure S2). The excessive appearance of GFP likely results from cellular lysis caused by toxicity. Therefore, the four compounds with excessive GFP secretion were censored from the statistical analysis, leaving nine compounds with significant reduction and five demonstrating a formally significant but questionable increase (Figure 2c). The two blinded negative controls exhibited no significant effect on GFP secretion (green arrows, Figure 2c). To evaluate the observed increase in GFP secretion, we repeated the analysis with the subset of nine compounds that initially triggered excessive GFP secretion. Importantly, this subset also included the four compounds that were censored from the initial screening dataset. Interestingly, upon replication, the excessive response was not confirmed (Figure 2d). This further supports random, unspecific GFP delivery to the supernatant linked to unspecific toxicity but unlikely agonistic effects on Rab11. Thus, we focused on the nine compounds with significant inhibitory effects (Figure 2e).

### 2.3. Validation of Positive Hits and Evaluation of FIP3 Binding

**Figure 3:**
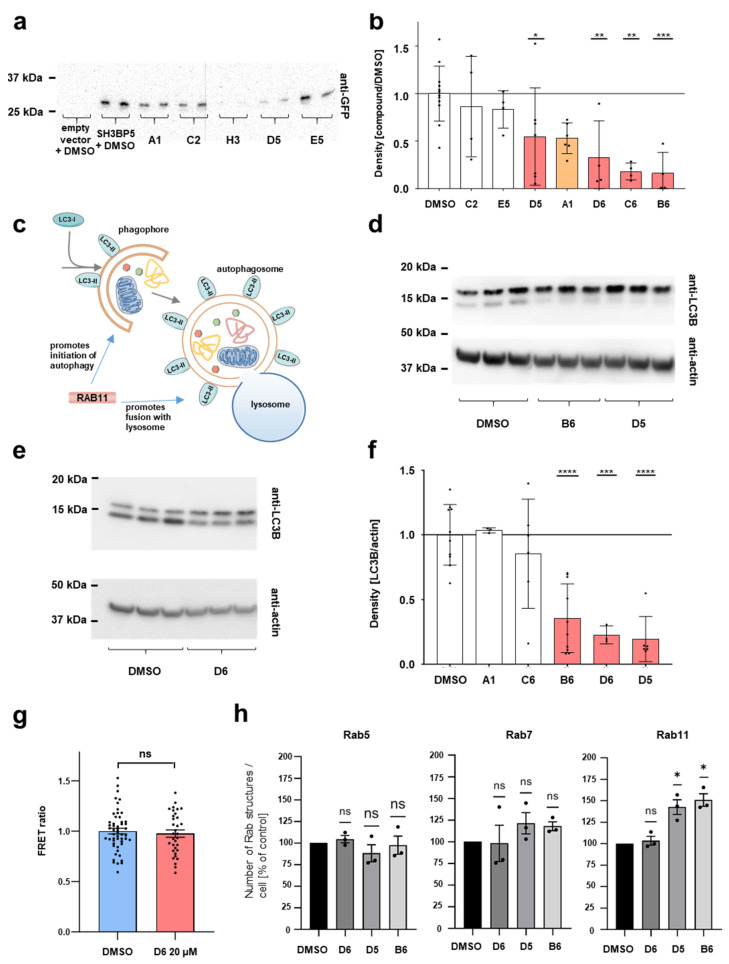
Validation for potency, by detection of basal autophagy and subcellular localization. (**a**) Representative Western blot stained for secreted GFP in the supernatant at a dose of 5 µM shows variable effects using the lower dose, which is compatible with variable potency. (**b**) Quantification of immunoblotting using anti-GFP antibody on supernatant from HEK293T cells stably expressing secretory GFP after compound exposure at 5 µM is shown for seven compounds. Two of the nine compounds significant at 20 µM were censored here due to an excessive response. At the lower dose, only compounds B6, C6, D5, and D6 showed a significant reduction, while A1 showed a trend that was not significant (mean ± SD, *n* = 4–7 per condition, *p* < 0.05 for D5, *p* < 0.01 for D6 and C6, *p* < 0.001 for B6. For the remaining compounds, *p* > 0.05). (**c**) Schematic illustrates phagophore formation with activation of LC3-I to LC3II, which marks the autophagosomes. The phagophore elongates to form the mature autophagosome, which in turn fuses with the lysosome. RAB11 promotes both phagophore formation and the lysosomal fusion event. (**d**,**e**) Immunoblotting of lysates from HEK293T cells after compound exposure using anti-LC3B reveals a lower band around 15 kDa that reflects the active LC3B that travels faster due to lipidation. Cells have been treated with 80 µM chloroquine for 2 h to show basal autophagic flux. Treatment with compounds B6 and D5 (**d**) and D6 (**e**) reduces the lower band that corresponds to LC3-II. (**f**) Quantification of density of LC3-II/loading control analogous to experiment in (**c**) is shown for the indicated genotypes. Compounds D5, B6, and D6 show a significant reduction in basal autophagy, suggesting an effect on the activity of RAB11 (mean ± SD, *n* = 3–10 per condition, *p* > 0.05 for A1 and C6, *p* < 0.001 for D6, and *p* < 0.0001 for B6 and D5). (**g**) Quantitative analysis of FRET efficiency (FRET ratio) is shown as readout of RAB11A-GTP loading after application of Rab11-inhibitor-D6 at 20 µM for 16 h compared to vehicle does not prevent GTP loading (mean ± SE, *n* = 53 cells for vehicle and 33 cells for Rab11-inhibitor-D6 condition, *p* > 0.05). (**h**) Quantification of the number of Rab5 (left panel), Rab7 (middle panel), and Rab11 (right panel) vesicles per single Cos-7 cell in the peripheral region when treated with either DMSO (vehicle) or D6 or D5 or B6 compounds. Error bars represent mean ± S.E.M. n.s., not significant, *p* < 0.05 (one-sample Student’s *t*-test), *n* = 3 independent experiments.

To identify strong candidates for further analysis, we first evaluated the nine significant hits for their potency. We repeated the screening assay with secretory GFP for the nine remaining candidates, this time reducing the dose from 20 µM to 5 µM to test their potency. Two compounds showing a mildly increased response in the lower exposure were excluded from further analysis (Supplementary Figure S3a). With the lower dose, only four compounds indicated significant inhibition. One compound (A1) showed a strong, though non-significant, reduction in GFP secretion at the lower dose (representative membrane Figure 3a, quantification Figure 3b). We focused on the five most potent candidates for further validation. Our primary screening assay reflects the activity of RAB11 indirectly using exocytosis. Given the possibility that a small molecule might inhibit exocytosis directly but without affecting RAB11 function, we wanted to examine a cellular process that requires RAB11 but not exocytosis. Therefore, we measured basal autophagy. RAB11 promotes both the early stage of phagophore formation and the late stage of fusion of autophagosomes and with lysosomes [6,8] (Figure 3c). To detect autophagy, we used the autophagy marker LC3B, which undergoes cleavage and lipidation to obtain the active form LC3-II locating on autophagosomes (Figure 3c). The ratio of the lower of two bands to a loading control reflects active autophagosomes and the inhibition of lysosomal degradation by chloroquine reveals autophagic flux [31]. Without chloroquine, we observed only minor differences using the candidate compounds that became more pronounced by the use of chloroquine (Supplementary Figure S3b). This suggests a decrease in autophagic flux, which is consistent with a decrease in RAB11 function [13]. We tested the five candidates for an effect on autophagy at 20 µM (representative membrane Figure 3d,e, quantification Figure 3f). Two of the potent compounds had no impact on autophagy, suggesting their impact on GFP secretion may be independent of RAB11 (Figure 3f). However, we observed a significant reduction for three compounds (D5, B6, and D6, Figure 3f). Since these small molecules inhibit both exocytosis and autophagy, at least a partial inhibitory effect on RAB11 seemed highly likely. Consequently, we named the substances Rab11-inhibitor-D5, Rab11-inhibitor-B6, and Rab11-inhibitor-D6.

Next, we wanted to examine if our inhibitors directly interfere with the recruitment of effector FIP3. We tested Rab11-inhibitor-D6 utilizing a biosensor based on Fluorescence Resonance Energy Transfer (FRET) [32]. In this probe, RAB11A is fused to cyan (mECFP)- and yellow (mcpVenus)-emitting fluorescent proteins, together with the C-terminal portion of *RAB11FIP3*, a RAB11 effector that recognizes the GTP-bound RAB11 form, specifically. GTP loading of RAB11A promotes interaction with FIP3, bringing the probe’s two fluorescent proteins into closer proximity. This in turn increases FRET efficiency and concomitantly decreases anisotropy-based FRET. However, in testing Rab11-inhibitor-D6, we did not observe a difference in FRET efficiency or anisotropy (Figure 3g and Figure S3c,d). Recruitment of effector FIP3 therefore seems undiminished, indirectly suggesting that RAB11-GTP loading itself is not prevented by Rab11-inhibitor-D6. This suggests that the inhibitory role may be incomplete. However, this outcome does not negate an overall inhibitory role. In our FRET assay, the C-terminal section of FIP3 interacts with multiple sections of RAB11 simultaneously [29]. Therefore, blocking a single FIP3 interaction site on RAB11 may not suffice to prevent FRET activity. The efficacy of our small molecule, validated by two independent downstream readouts, may also be contingent on effector proteins that are unrelated to the effector FIP3. To evaluate a specific impact on Rab11 independently, we studied the subcellular localization of endogenous Rab proteins in Cos7 cells. Upon treatment with Rab11-inhibitor-D5 and B6 but not D6, we noted a significant increase in the numbers of Rab11 vesicles as well as a shift in Rab11 towards the cell periphery while Rab5 and Rab7 were unaffected (Figure 3h and Figures S4 and S5a). This suggests an impact specifically on Rab11 transport. Size and fluorescence intensity were not altered (Supplementary Figure S5b,c). This further supports an inhibitory effect for Rab11-inhibitor-B6 and Rab11-inhibitor-D5, while D6 requires further analysis.

### 2.4. Dose-Response Analysis Reveals a Bell-Shaped Response with Potential Hormesis

**Figure 4:**
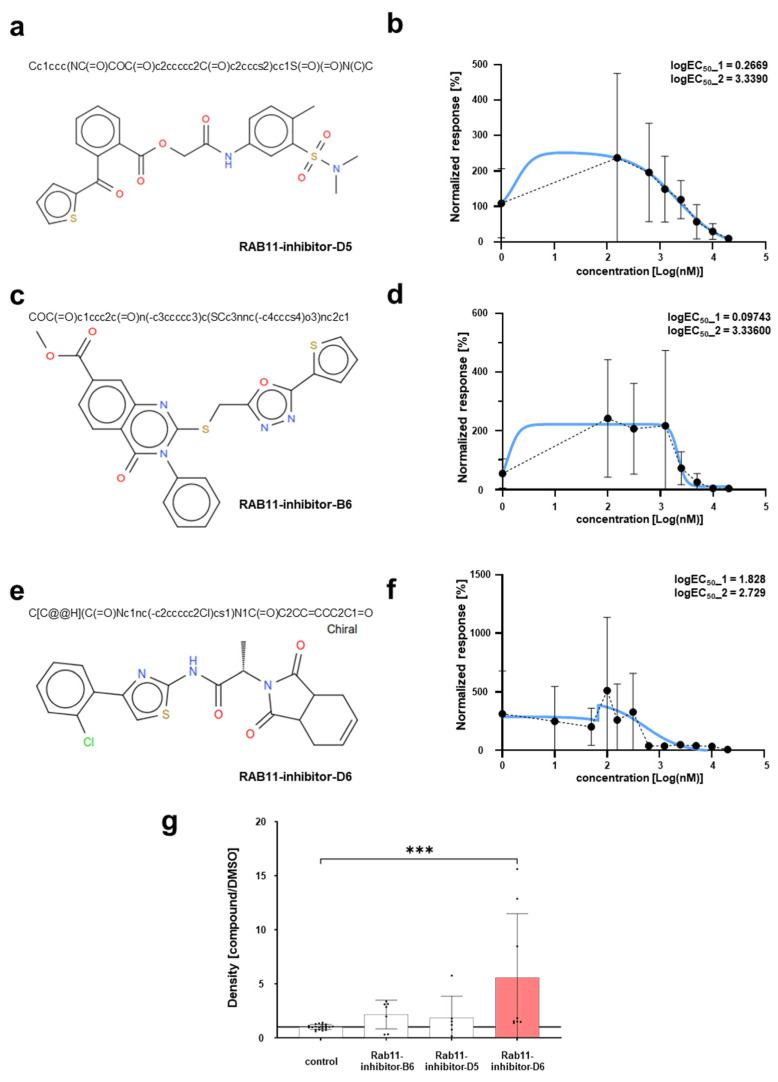
Dose–response relationship indicates a hormetic response. (**a**) Shown is the structure and Simplified Molecular Input Line Entry System (SMILES) string of RAB11-inhibitor D5. (**b**) The dose–response curve illustrates a bell-shaped relationship between increasing doses of an RAB11-inhibitor-D5 treatment and the corresponding changes in GFP secretion. GFP was evaluated by densitometry after immunoblotting with anti-GFP using our secretory GFP HEK293T cells. The y-axis shows the normalized response of treated cells against the vehicle (DMSO) in percent that is plotted against the dose range in logarithmic scale on the x-axis. The data were fitted using non-linear regression for a biphasic, bell-shaped effect (blue line), with an R-squared value of 0.43. The logEC_50_ values were 3.34 µM and 0.27 µM for Rab11-inhibitor-D5 (*n* = 2–5 per concentration). (**c**) Shown is the structure and Simplified Molecular Input Line Entry System (SMILES) string of RAB11-inhibitor B6. (**d**) The dose–response curve illustrates the biphasic relationship between increasing doses of an RAB11-inhibitor-B6 treatment and the corresponding changes in GFP analogous to (**b**). The data were fitted using non-linear regression for a biphasic, bell-shaped effect (blue line), with an R-squared value of 0.45. The logEC_50_ values were determined as 3.34 µM and 0.10 µM for Rab11-inhibitor-B6 (*n* = 2–5 per concentration). (**e**) Shown is the structure and Simplified Molecular Input Line Entry System (SMILES) string of RAB11-inhibitor D6. (**f**) The dose–response curve shows a largely biphasic relationship between increasing doses of an RAB11-inhibitor-D6 treatment and the respective changes in GFP analogous to (**b**). The data were fitted using non-linear regression for a biphasic effect (blue line), with an R-squared value of 0.30. The resultant logEC_50_ values were 2.73 and 1.83 µM for Rab11-inhibitor-D6 (*n* = 2–5 per concentration). (**g**) Quantification of immunoblotting using anti-GFP antibody on supernatant from HEK293T cells stably expressing secretory GFP after compound exposure at 1.25 µM is shown. There is a trend towards increased secretion that is statistically not significant for RAB11-inhibitor-B6 and RAB11-inhibitor-D5 and a statistically significant increase for RAB11-inhibitor-D6 (mean ± SD, *n* = 6–8 per genotype, *p* > 0.05 for RAB11-inhibitor-B6 and RAB11-inhibitor-D5, *p* < 0.001 for RAB11-inhibitor-D6).

To explore the efficacy of our inhibitors, we studied the dose–response relationship of Rab11-inhibitor-D5, Rab11-inhibitor-B6, and Rab11-inhibitor-D6 at a dose range from 1 nM to 20 µM using the secretory GFP assay (shown together with the respective structures, Figure 4a–f). For each of the inhibitors, the curve did not display a classical sigmoidal shape, which is characterized by a plateau at lower dosages. Instead, a biphasic, roughly bell-shaped dose–response curve was noted. While high doses effectively and dose-dependently inhibit GFP secretion, a lower dosage exhibited a fairly variable response that was partly well in excess of the control level. A biphasic response may indicate hormesis, where low doses elicit a paradoxical gain-of-function, while higher doses produce an inhibitory effect. We applied a linear regression designed for the bell-shaped, biphasic response using GraphPad Prism 9.3.1 software and calculated two logEC_50_ values for both aspects of each compound. The values were 3.34 µM and 0.27 µM for Rab11-inhibitor-D5, 3.4 µM and 0.10 µM for Rab11-inhibitor-B6, and 2.73 and 1.83 µM for Rab11-inhibitor-D6. The precision of the lower logEC_50_ values regarding the stimulatory response is limited due to the variability in the dose–response relationship at the intermediate range, while the inhibiting response at higher doses was much more robust (Figure 4b,d,f). We evaluated the results of a single low dose in isolation, and we determined a significant effect for Rab11-inhibitor-D6 and a trend for the other two novel Rab11 inhibitors that was not significant (Figure 4g). A hormetic effect thus may exist for at least one of the inhibitors.

### 2.5. The Novel RAB11-Inhibitors Exhibit Low Toxicity and Good Druglikeness

Next, we wanted to examine the basic properties and toxicity of the novel inhibitors regarding their role as putative drug candidates. We first estimated the druglikeness of our validated inhibitors using an in silico open access tool [33]. While not representing definitive indicators of drug potential, druglikeness scores comprise computational metrics regarding physicochemical properties and structural features that are desirable for drug development. According to the commonly used prediction score by Lipinski [34], all inhibitors show good drug potential (Table 1). The alternative set of Ghose and colleagues [35] similarly supports Rab11-inhibitor-B6 and Rab11-inhibitor-D6 but shows a single, and only marginal, violation for the molecular-weight Rab11-inhibitor-D5. The pharmacokinetic prediction suggests low oral bioavailability for Rab11-inhibitor-B6 and Rab11-inhibitor-D5 but high likelihood of gastrointestinal absorption for Rab11-inhibitor-D6. Passage of the blood–brain barrier is estimated as unlikely for all three inhibitors. We conclude that the chemical properties of our inhibitors are favorable for further investigation.

**Figure 5:**
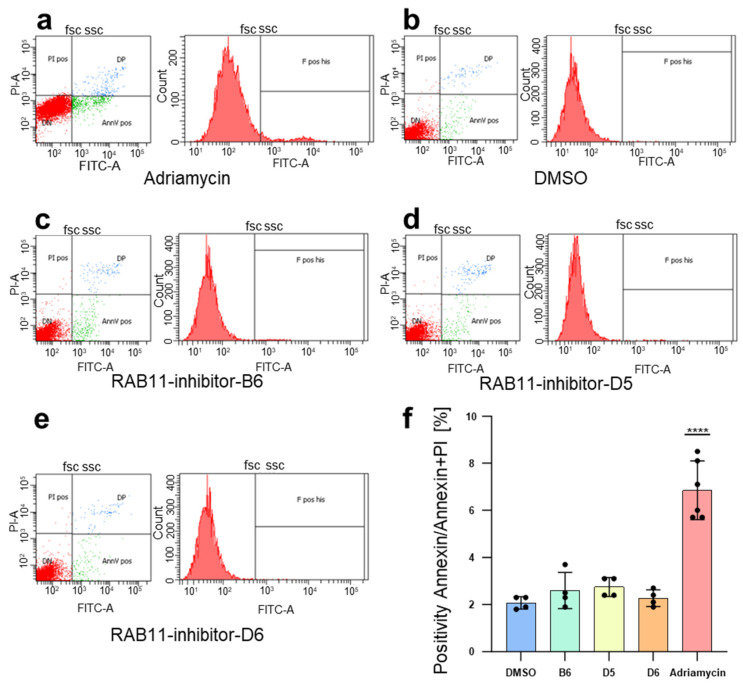
RAB11-inhibitors show no significant toxicity in vitro. (**a**–**e**) Immortalized podocytes were exposed to the respective compounds each at a concentration of 20 µM for 24 h preceding Annexin V/propidium iodide exposure and flow cytometry. Representative original dot plots (left) are shown for the indicated conditions. Green fluorescence indicates FITC-Annexin (apoptotic cells bottom right section in green) while red fluorescence represents propidium iodide (dead cells, upper left section in red). Cells negative for either cell death marker (bottom left section in red) or double positive cells (upper right section in blue) are shown as well. Corresponding histograms for green fluorescence under these conditions are displayed on the right. Elevated Annexin positivity compared to control (**b**) is observed for Doxorubicin (Adriamycin, panel a), but not for the novel inhibitors of RAB11 (**c**–**e**). (**f**) Quantification of data analogous to a-e (mean ± SD, *n* = 4, *p* < 0.0001 for Adriamycin compared to control, *p* > 0.05 for all RAB11-inhibitors).

To assess the potential toxicity of the novel Rab11 inhibitors identified in our study, we used cultured podocytes in vitro. These cells were chosen as a likely target cell for RAB11-associated kidney disease [13,14]. Each of the inhibitors was applied for 24 h on the immortalized podocytes at a dose of 20 µM. Cell death was determined using Annexin V binding and propidium iodide. Flow cytometry detected minimal, and not significantly, elevated toxicity as indicated by elevated Annexin binding or propidium iodide entry compared to the negative control (vehicle) at 20 µM (Figure 5a–f). In contrast, Doxorubicin (Adriamycin), a chemotherapeutic drug that served as the positive control, exhibited significantly higher toxicity (Figure 5f). Necrotic cell death as indicated by isolated positivity for PI was extremely low for all conditions (Supplementary Figure S6). In summary, we did not detect relevant toxicity at a pharmacologically highly effective dose of 20 µM.

### 2.6. Drosophila Studies Confirm Low Toxicity and Reveal Mislocalization of Drosophila Rab11

To examine the effects of our compounds in an in vivo model, we exposed *Drosophila melanogaster* to the novel inhibitors. This animal model harbors podocyte-like cells, the nephrocytes that can be used to study mechanisms of nephrotic syndrome with a hereditary, monogenic origin [36].

**Figure 6:**
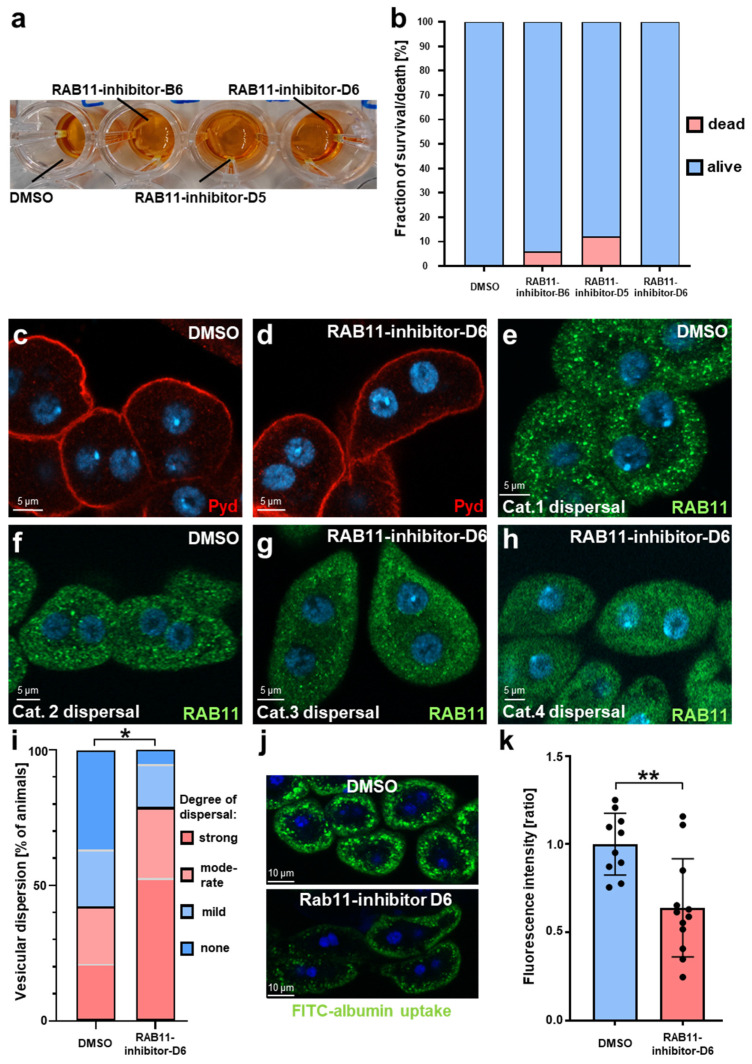
Drug exposure in *Drosophila* larvae confirms low toxicity and Rab11 inhibition by morphological and functional criteria. (**a**) Representative image showing *Drosophila* third instar larvae from a *Drosophila* strain similar to wild-type (yw^1118^) that are being exposed to RAB11-inhibitors or vehicle (DMSO) using 96-well plates and liquid food. (**b**) Quantification of surviving larvae after 24 h of drug exposure analogous to (**a**) as indicated by movement and feeding upon inspection, (*n* = 100 larvae per genotype). (**c**,**d**) Confocal images of garland cell nephrocytes stained for the slit diaphragm protein Pyd are shown after RAB11-inhibitor-D6 feeding (**d**) or DMSO control (**c**). (**e**–**h**) Representative confocal images of nephrocytes stained for RAB11 show increasing dispersal of Rab11 upon drug feeding and reflect four categories used for quantification (1: strong vesicular signal, low background, 2: strong vesicular signal, high background, 3: weak vesicular signal high background, 4: high background only). (**i**) Blinded quantification of data analogous to e-f using Chi-squared test (comparing two groups, categories 1 + 2 vs. 3 + 4) indicates a strong shift towards dispersal of RAB11 upon exposure to RAB11-inhibitor-D6 (*n* = 20 animals per genotype, *p* < 0.05). (**j**) FITC-albumin endocytosis as an assay of nephrocyte function is shown after exposure for 30 s and wash out of 5 min. Exposing larvae to Rab11-inhibitor-D6 in liquid food for 24 h strongly reduces uptake of FITC-albumin compared with the control (DMSO). (**k**) Quantification of results as average of the three brightest individual cells per animal from (**j**) in ratio to a control experiment performed in parallel (mean ± SD, *n* = 10–12 animals per genotype, *p* < 0.01 for exposure with Rab11-inhibitor-D6).

We first assessed toxicity in this animal model and fed the compounds to third instar *Drosophila* larvae. The animals were reared in 96-well plates containing liquid food with the addition of the Rab11-inhibitors at a dose of 50 µM or equal volumes of DMSO (Figure 6a). Around 90% of the animals survived over a period of 24 h in liquid food for all compounds (Figure 6b). Long-term exposure in liquid food is harmful to larvae even without any additives and thus cannot be studied. Interestingly, we observed a survival rate of 100% for RAB11-inhibitor-D6, which is predicted to exhibit the best oral bioavailability (Figure 6b).

We focused on RAB11-inhibitor-D6 due to its predicted oral bioavailability and studied the slit diaphragm architecture after drug exposure. We observed a regular staining pattern of the slit diaphragm protein polychaetoid (Pyd) comparing DMSO and Rab11-inhibitor-D6 (Figure 6c,d). This is in line with short-term gene silencing of *Drosophila* Rab11, where phenotypic effects on the slit diaphragm manifested after longer exposure [13] and further supports low toxicity. To examine an impact on the subcellular distribution of RAB11, we compared the staining pattern of endogenous Rab11 after exposure to Rab11-inhibitor-D6 for 24 h with DMSO alone. We observed a dispersal of the antibody signal from brighter vesicles to a more even distribution in the cytosol, which is compatible with an inhibitory effect on the *Drosophila* Rab11 within these cells (Figure 6e–h). A blinded analysis of this dispersal phenotype confirmed a significant shift in Rab11 towards the cytosol (Figure 6i). This suggests a reduced ability to form stable effector complexes on cellular membranes. Unlike the findings using this compound in cultured cells, we thus noted an induced mislocalization of Rab11 in this animal model. This provides further support for an inhibitory role. In contrast, the staining pattern of Rab7 appeared unchanged (Supplementary Figure S7a,b), supporting a specific effect. The overall intensity of the Rab11 staining across the entire cell remained unaltered (Supplementary Figure S7c–e). This supports a specific effect on the functional state of Rab11 in vivo. To explore impact on nephrocyte function, we determined FITC-albumin endocytosis, which is known to decrease significantly upon silencing of *Rab11* [12]. Accordingly, we observed a significant reduction in nephrocyte function upon exposure to Rab11-inhibitor-D6 in comparison to the vehicle control (Figure 6j,k). This is further consistent with the in vivo inhibition of RAB11 upon exposure to the inhibitor.

In summary, we identify three small molecules as inhibitors of Rab11 in a tiered screening approach combining the preselection of candidates using artificial intelligence with a platform based on exocytosis with validation using autophagy. We explore druglikeness and validate an impact on Rab11 in an in vitro setting and in *Drosophila*.

## 3. Discussion

RAB11 plays an important role for a range of cellular functions and has been implicated in human disease [5,13,17,18,32]. However, no direct inhibitor for this protein has so far been discovered, since large-scale screening is difficult. To address this challenge, we applied a synergistic screening approach. Firstly, we established an assay to monitor the activity of endogenous Rab11 by the detection of RAB11-dependent exocytosis. Combining deep learning-based virtual screening with our platform, we identified a set of 94 potential candidates in silico and then confirmed 9 hits among these in vitro. From these, we selected compounds with greater potency and validated three of them using basal autophagy, as an independent cellular function that relies on RAB11. We examined the dose–response relationship of three compounds and discovered a biphasic, potentially hormetic response. All three inhibiting compounds show drug-like properties, making them good candidates for drug development. Using subcellular distribution of Rab11 in cells and the *Drosophila* model, we confirmed an impact on Rab11 localization. Both models further showed an absence of relevant toxicity. In addition to being potential candidates for Rab11-associated disease, the newly discovered inhibitors can be immediately useful for scientific investigation.

The challenge to definitively determine the functional state of RAB11 is an inherent limitation of this study. While we cannot provide definitive proof of an inhibitory role, a specific modulation of Rab11-dependent function for these compounds is strongly supported by our data. The observation of an impact on two unrelated cellular functions that each depend on RAB11 is a strong indication of an effective inhibitor for the three compounds. Importantly, this is further supported by the mislocalization of Rab11 in Cos7 cells or *Drosophila* nephrocytes and the decrease in nephrocyte function in vivo. Using a FRET biosensor [32], we noted the recruitment of FIP3 despite Rab11-inhibitor-D6. This suggests that GTP loading of RAB11 is not prevented and partial function may be retained. Impaired recruitment of other effector proteins might explain the functional decline that we observed. Global knockout of the murine Rab11a gene resulted in embryonic lethality [37]. A selective modulatory effect therefore may be more desirable. Pharmacological attenuation of RAB11 function may be beneficial in specific disease settings since several recycling pathways exist that may partially compensate in later stages of life [38,39]. The observation that our presumptive inhibitors altered Rab11 localization, while neither affecting Rab5 nor Rab7 in Cos7 cells or Rab7 in nephrocytes, suggests a certain degree of specificity for Rab11. However, further studies are required to exclude potential off-target effects.

The nature of the biphasic dose–response of the inhibitors remains an open question. Hormesis has not been specifically described for Rab11, but this phenomenon is frequently observed in biological systems [40]. While the mechanistic basis remains poorly understood, hormesis has been linked to the excessive function of a partially inhibited multimer [41]. We speculate that at lower doses, the partial inhibition of RAB11 multimers may result in a net activation. The effect reverts to the contrary, once increasing the dosage of the inhibitor blocks RAB11 entirely.

The structural similarity between the effective compounds identified in the screen is intentionally low, as the screen was designed to identify high-ranking compounds with diverse structures. This design aims to maximize the likelihood of finding effective substances. For this reason, we did not analyze the structure–activity relationship. 

The compounds may be a useful tool for research purposes. These inhibitors close a relevant gap to dissect pathways of endocytosis, exocytosis, and autophagy, which are intricately linked with a wide array of signaling pathways. Our study finally represents a successful example of how drug discovery is likely to be reshaped by the application of artificial intelligence for computational drug discovery in the near future [19].

## 4. Materials and Methods

### 4.1. The Plasmids, Cell Culture, and Transfection

Overexpression and immunoblotting were performed in human embryonic kidney (HEK293T) cells. HEK293T or Cos-7 cells were maintained at 37 °C in DMEM, supplemented with 10% fetal bovine serum, 50 IU/mL penicillin, and 50 μg/mL streptomycin. Lipofectamine 2000 (Invitrogen, Darmstadt, Germany) was used for transfection into HEK293T cells. For chloroquine treatment, cells were exposed to culture medium containing 80 µM chloroquine for 2 h.

SH3BP5 was derived from Addgene (Watertown, MA, USA) plasmid #23579, a gift from William Hahn & David Root, and cloned into pcDNA6.2-N-GFP with the tag in frame using PCR and Gibson DNA Assembly (New England Biolabs, Frankfurt, Germany). We previously described an assay for RAB11 activity by introduction of the signal peptide of IFNA2 into the N-terminus of GFP (pCDNA6.2-C-GFP). To obtain stable transduction, this cassette was amplified by PCR (Phusion polymerase, New England Biolabs, Frankfurt, Germany) and digested with MluI-HF and NotI-HF for transfer into the pLXSN vector. For lentivirus production, the modified pLXSN was transfected together with pMD-g (envelope) and pMD-g/p (packaging construct encoding for gag and pol) into HEK293T cells using lipofectamine. The virus-containing cell medium (supernatant) was applied on HEK293T cells, and single cellular clones were selected for strongest expression of GFP in the cellular supernatant.

### 4.2. Virtual Compound Screening

Atomwise used their proprietary AtomNet^®^ platform, a deep convolutional neural network for structure-based drug design [23,42,43,44], to perform a virtual high-throughput screen of 2.27 million compounds from the Enamine in-stock small-molecule library (version 20200204, https://enamine.net/ accessed 12 October 2024), as described previously [25,26]. The top 30,000 predicted compounds were filtered for undesired chemical moieties and physicochemical properties (clogP ≤ 5, number of rotatable bonds ≤ 8, number of unspecified stereocenters ≤ 2) followed by ECFP4 fingerprint-based Butina clustering with a Tanimoto coefficient of 0.35 for similarity cutoff. From this list, the top 94 compounds were purchased and supplied as 10 mM stock solutions in DMSO, together with two DMSO negative controls. The compounds were blinded by the vendor for the biological screening and the compound structures were revealed after the primary assay data were generated. Rab11 inhibitors are available from Enamine (IDs: Z66498322, Z17937228, and Z57011850).

### 4.3. Immunoblotting

For immunoblotting, cells of the respective conditions were lysed by incubation for 15 min in ice-cold immunoprecipitation lysis buffer containing protease inhibitor (Merck/Roche, Darmstadt, Germany) followed by sonication. The extracts were centrifuged for 15 min at 14,000 rpm and protein concentration was determined using a colorimetric assay (Bio-Rad, Feldkirchen, Germany). For detection of secreted GFP, the undiluted supernatant was loaded directly without measurement of protein concentration. Samples were heated to 72 °C for 10 min and loaded on a 4–12% SDS–polyacrylamide gel for electrophoresis. Protein was transferred to polyvinylidene difluoride membranes (Millipore/Thermo Fisher Scientific, Darmstadt, Germany). For detection of GFP-tagged constructs or GFP within the cellular supernatant, a mouse anti-GFP antibody was applied (1:1000, Santa Cruz, sc-9996, Heidelberg, Germany). Mouse anti-actin (1:1000, JLA20, DSHB) or rabbit anti-GAPDH (1:1000, Cell Signaling Technologies, 2118S, Leiden, The Netherlands) served as loading control. Rabbit anti-LC3B (1:1000, 2775; Cell Signaling Technology, Leiden, The Netherlands) was used to detect basal autophagy.

### 4.4. FRET Biosensor Analysis and Subcellular Rab Analysis

To evaluate FRET activity, we applied an established biosensor pair [32]. Cos-7 cells were grown on a 12 mm (No 1.5H) microscope cover glass. Cells were transfected using Lipofectamine 3000 (Invitrogen) according to the manufacturer’s instructions. After 6 h from transfection, cells were treated with Rab11 inhibitor. After 24 h post-transfection and 16 h post-Rab11 inhibitor treatment, cells were fixed by 4% PFA for 15 min at room temperature. FRET images were acquired on an inverted confocal Leica SP8 microscope equipped with a 40x Oil immersion objective, NA 1.25. Hyd detectors (Leica, Wetzlar, Germany) allowed the simultaneous detection of mECFP and mcpVenus. Fluorescent dyes were imaged sequentially in frame-interlace mode to eliminate crosstalk between the channels. mECFP was excited with a 458 nm laser line and imaged through 470–488 nm and 520–540 nm bandpass emission filters. Monomeric circular permutated Venus (mcpVenus) was excited with a 488 nm laser line and imaged through a 500–540 nm bandpass emission filter. Images were processed and analyzed by Leica Application Suite X (LAS X) software (v3.7.6.25997).

For anisotropy-based FRET, Hek293T cells were grown in 6-well plates and transfected using Polyethylenimine (PEI) with a ratio DNA (μg):PEI of 1:2.5. After 36 h post-transfection, cells were lysed in lysis buffer (10 mM Tris–HCl, pH 7.4, 10 mM MgCl_2_, 100 mM NaCl, 1% Triton^TM^ X-100, Merck/Sigma, Milan, Italy, proteinase inhibitors). Rab11 inhibitor was added to the clarified lysates and incubated for 5 h with gentle agitation. Lysates were excited at 430 nm and emission was measured at 535 nm. Anisotropy was measured using Spark Multimode Microplate Reader (TECAN, Männedorf, Switzerland) instrument.

For analysis of subcellular distribution of endogenous Rab proteins, Cos7 cells were exposed to compounds or DMSO before detection of endogenous Rab5, Rab7, and Rab11 via immunofluorescence using anti-Rab5 (BD Lab, Florence, Italy, 610725), anti-Rab7 (Cell Signaling Technology, Leiden, The Netherlands, # 9367), and anti-Rab11 (Cell Signaling Technology, Leiden, The Netherlands, #5589). Cells were imaged using a Leica SP8 confocal microscope. The number, area, mean intensity, and the distance from the nucleus for each Rab protein were analyzed using MATLAB R2023b and CellProfiler software (v4.2.5).

### 4.5. Drosophila Studies

Flies were reared on standard food at 25 °C. To estimate toxicity in living animals and the impact on subcellular Rab11 distribution, we collected third instar larvae from a wild-type variant (yw^1118^), which were exposed to Rab11-inhibitor-D5, Rab11-inhibitor-B6, and Rab11-inhibitor-D6 for 24 h. The compounds were dissolved in a dimethyl sulfoxide (DMSO) stock solution at 10 mM and diluted in liquid Drosophila food (H_2_O with 5% sucrose, 10% yeast extract, and 0.5% propionic acid) to a final concentration of 50 µM. DMSO at 0.5% in liquid food served as negative control. Larvae were treated in 96-well plates with a maximum of six larvae per well.

For immunofluorescence, nephrocytes from treated larvae were dissected, fixed for 20 min in phosphate-buffered saline containing 4% paraformaldehyde, and blocked in 5% albumin for 1 h before incubation in the following primary antibodies overnight: rabbit anti-RAB11 (#5589S; Cell Signaling Technology), mouse anti-pyd (PYD2, Developmental studies hybridoma bank [DSHB]), or mouse anti-Rab7 (RAB7, DHSB). Alexa fluorophore-conjugated secondary antibodies (Invitrogen) were applied for 2 h before mounting in ROTI-Mount (Carl Roth, Karlsruhe, Germany). Nuclei are marked in blue by Hoechst 33342 throughout the manuscript.

To evaluate nephrocyte function, we applied FITC-albumin ex vivo in a modification of a previously established protocol [36]. Briefly, after dissection in PBS nephrocytes were incubated with FITC-albumin (Merck/Sigma, Darmstadt, Germany) for 30 s. Cells were rinsed four times and incubated for another five minutes to ensure tracer reached the endosomes and unbound tracer was washed out from the extensive network of membrane invaginations in nephrocytes. After a fixation step of 5 min in 8% paraformaldehyde containing Hoechst 33342 (1:1000), cells were rinsed in PBS and mounted in Roti-Mount (Carl Roth). Cells were imaged using a Zeiss LSM 980 laser scanning microscope (Oberkochen, Germany). Quantification of fluorescent tracer uptake was performed with ImageJ software (2.14.0) for the brightest three cells per animal. The results are expressed as a ratio to a control experiment performed in parallel.

### 4.6. Annexin Measurement in Cultured Podocytes

Immortalized human podocytes were a gift from Dr. Moin Saleem (University of Bristol, Bristol, UK). Cells were grown at the permissive temperature of 33 °C and maintained in RPMI 1640 GlutaMAX (Thermo Fisher Scientific) supplemented with 10% FBS, 50 IU/mL penicillin, 50 μg/mL streptomycin, and Insulin-Transferrin-Selenite Supplement (Roche/Sigma 11074547001). To determine the type and extent of cell death after an exposure to the indicated compounds for 24 h, we used an FITC Annexin V Apoptosis Detection Kit (Biolegend, Amsterdam, The Netherlands, 640914) according to the manufacturer’s instructions. Briefly, podocytes were trypsinized, washed in cell staining buffer (BioLegend, 420201), and resuspended in 100 µL Annexin V Binding Buffer before adding 5 µL FITC Annexin V and 5 µL propidium iodide for 15 min. Cell death was determined after adding another 400 µL Annexin V Binding Buffer by flow cytometry (LSR Fortessa, Becton Dickinson Biosciences, Heidelberg, Germany).

### 4.7. Statistics

Unpaired *t* test was used to test for statistical significance between two groups. One-way ANOVA followed by Dunnett’s correction for multiple testing (unless otherwise indicated) was used for multiple comparisons. Measurements were from distinct samples and were tested for Gaussian distribution. Chi-squared test was applied for contingency tables. All statistic tests were performed using GraphPad Prism software. Asterisks indicate significance as follows: * *p* < 0.05, ** *p* < 0.01, *** *p* < 0.001, **** *p* < 0.0001. A statistically significant difference was defined as *p* < 0.05 unless otherwise indicated. Error bars indicate standard deviation (SD) unless specified otherwise.

## Figures and Tables

**Table 1:** Prediction scores for druglikeness. Red color indicates violation of the Lipinski or Ghose druglikeness rules, green marks compliance.

Lipinski Rule	Weight	Lipophilicity	H-Bond Acceptors	H-Bond Donors	Violations
Cut-Off	MW < 500	cLogP < 5	HBA < 10	HBD < 5	
Rab11-inhibitor D5	486.56 g/mol	3.22	7	1	0
Rab11-inhibitor B6	476.53 g/mol	4.11	7	0	0
Rab11-inhibitor D6	415.89 g/mol	3.07	4	1	0
**Ghose Filter**	**Weight**	**Lipophilicity**	**Molar Refractivity**	**Atoms**	**Violations**
**Cut-Off**	**MW 180–480**	**−0.4–+5.6**	**40–130**	**20–70**	
Rab11-inhibitor-D5	486.56 g/mol	4.22	125	33	1
Rab11-inhibitor-B6	476.53 g/mol	4.42	126	33	0
Rab11-inhibitor D6	415.89 g/mol	3.17	112.64	28	0

## Data Availability

No large-scale datasets from high-throughput analyses were generated or analyzed in this study. Unprocessed confocal images or immunoblots are available upon reasonable request to the corresponding author. All remaining data are included within the manuscript.

## Supplementary Materials

The following supporting information can be downloaded at: https://www.mdpi.com/article/10.3390/ijms252313224/s1.
