# Supplementary material for: Discovery of a Small Molecule with an Inhibitory Role for RAB11
Source: Int J Mol Sci. 2024 Dec 9;25(23):13224. doi: 10.3390/ijms252313224 (PMC11642393; doi:10.3390/ijms252313224)
Supplement: Supplementary file 1 [file ijms-25-13224-s001.zip › ijms-3330664-supplementary.pdf]

a

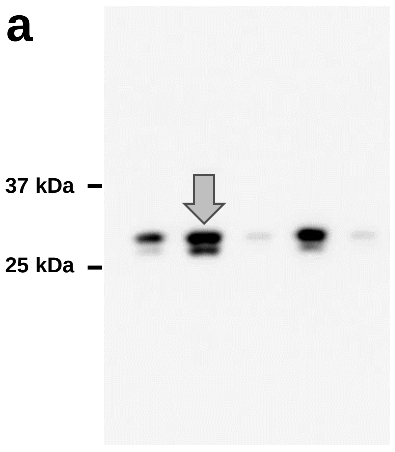

b

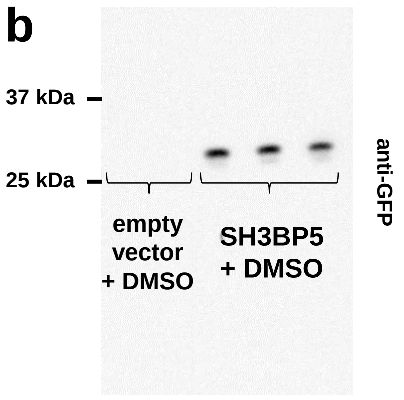

$$\text{Z-factor} = 1 - \frac{3(\sigma_p + \sigma_n)}{|\mu_p - \mu_n|}$$

c

RAB11A

MGTRDDEYDYLFKVVLIGDSGVGKSNLLSRFTRNEFNLESKSTIGVEFATRSIQVDGKTIKAQIWDTAGQERYRAITS
AYYRGAVGALLVYDIAKHLTYENVERWLKELRDHADSNIVIMLVGNKSDLRHLRAVPTDEARAFAEKNGLSFIETSAL
DSTNVEAAFQTILTEIYRIVSQKQMSDRRENDMSPSNNVVPIHVPPTTENKPKVQCCQNI

RAB11B

MGTRDDEYDYLFKVVLIGDSGVGKSNLLSRFTRNEFNLESKSTIGVEFATRSIQVDGKTIKAQIWDTAGQERYRAITS
AYYRGAVGALLVYDIAKHLTYENVERWLKELRDHADSNIVIMLVGNKSDLRHLRAVPTDEARAFAEKNNLSFIETSAL
DSTNVEEAFKNILTEIYRIVSQKQIADRAAHDESPGNNVVDISVPPTTDGQKPNKLQCCQNL

Supplementary Figure 1. Establishment of the screening platform.
(a) Immunoblot from supernatant of different clonal candidates for stably transduced HEK293T cells. Candidate #17 (arrow head) was chosen for the drug screen.
(b) Immunoblot of the same cell line as in (A) after transfection with SH3BP5 shows a strong signal after transient transfection with RAB11-GEF SH3BP5. Transfection with empty vector shows no signal in this relative range of detection. Empty vector serves as positive control (secretion with low endogenous activity of RAB11 comparable to an inhibitor) while SH3BP5 + DMSO serves as negative control (lack of inhibition of RAB11). The formula for Z-factor calculation is denoted on top.
(c) Protein sequence of RAB11A and RAB11B is shown in comparison. The residues targeted in the virtual screen are highlighted in red. The residue highlighted in yellow (A75) is occasionally denoted as A75R in the database, but the wildtype variant was used for the structure that is the basis of the screen[27].

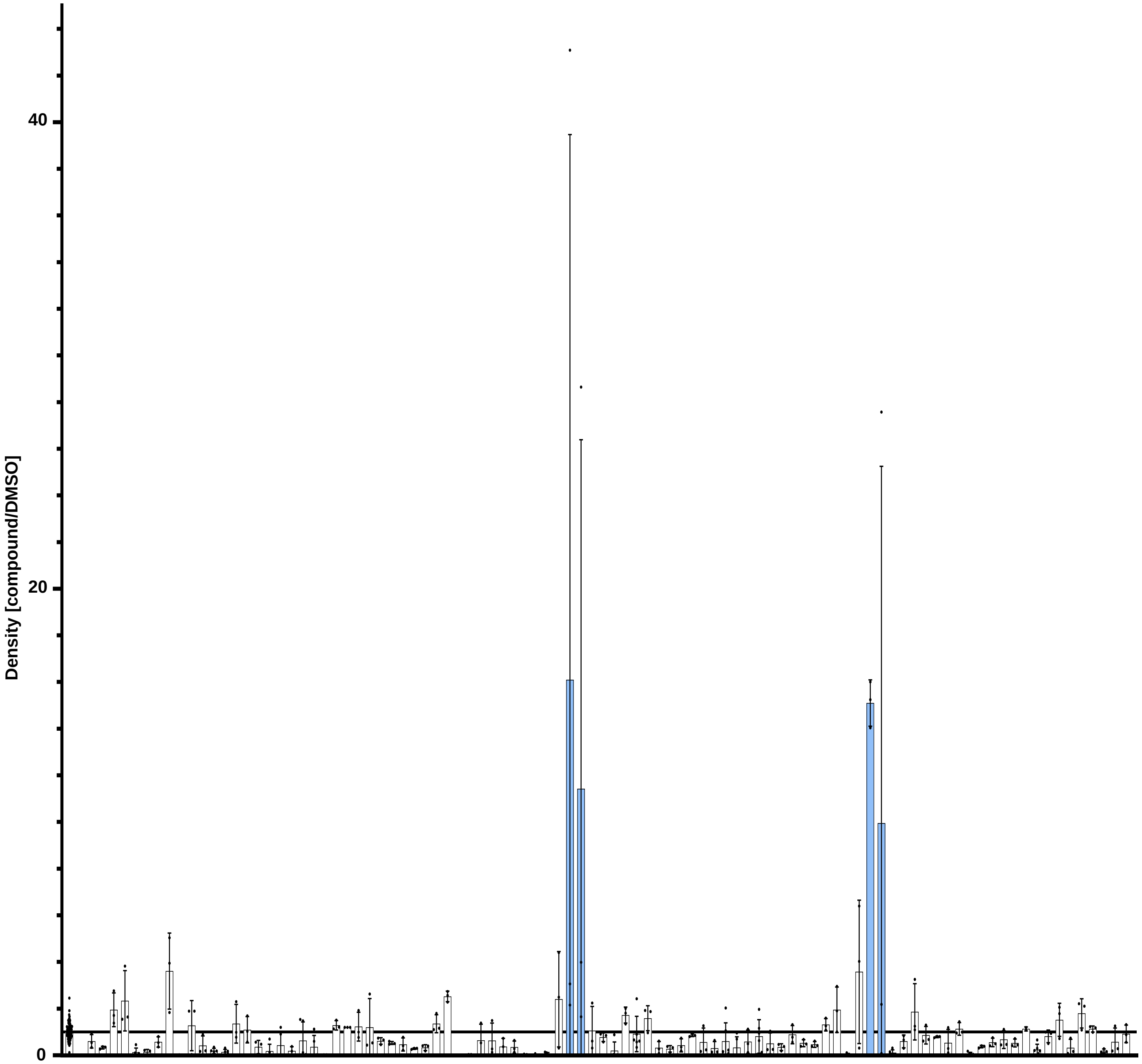

Supplementary Figure 2. Quantification of entire screening data.
Shown is the entire data set of the drug screen as Quantification of immunoblotting using anti-GFP antibody on supernatant from HEK293T cells stably expressing secretory GFP after compound exposure at 20 µM for 24 h normalized to control (n = 3-5 per condition). The severe excess of GFP secretion for some compounds (blue) make an interpretation difficult. Excessive secretion like reflects toxicity.
From left to right: A1, B1, C1, D1, E1, F1, G1, H1, A2, B2, C2, D2, E2, F2, G2, H2, A3, B3, C3, D3, E3, F3, G3, H3, A4, B4, C4, D4, E4, F4, G4, H4, A5, B5, C5, D5, E5, F5, G5, H5, A6, B6, C6, D6, E6, F6, G6, H6, A7, B7, C7, D7, E7, F7, G7, H7, A8, B8, C8, D8, E8, F8, G8, H8, A9, B9, C9, D9, E9, F9, G9, H9, A10, B10, C10, D10, E10, F10, G10, H10, A11, B11, C11, D11, E11, F11, G11, H11, A12, B12, C12, D12, E12, F12, G12, H12.

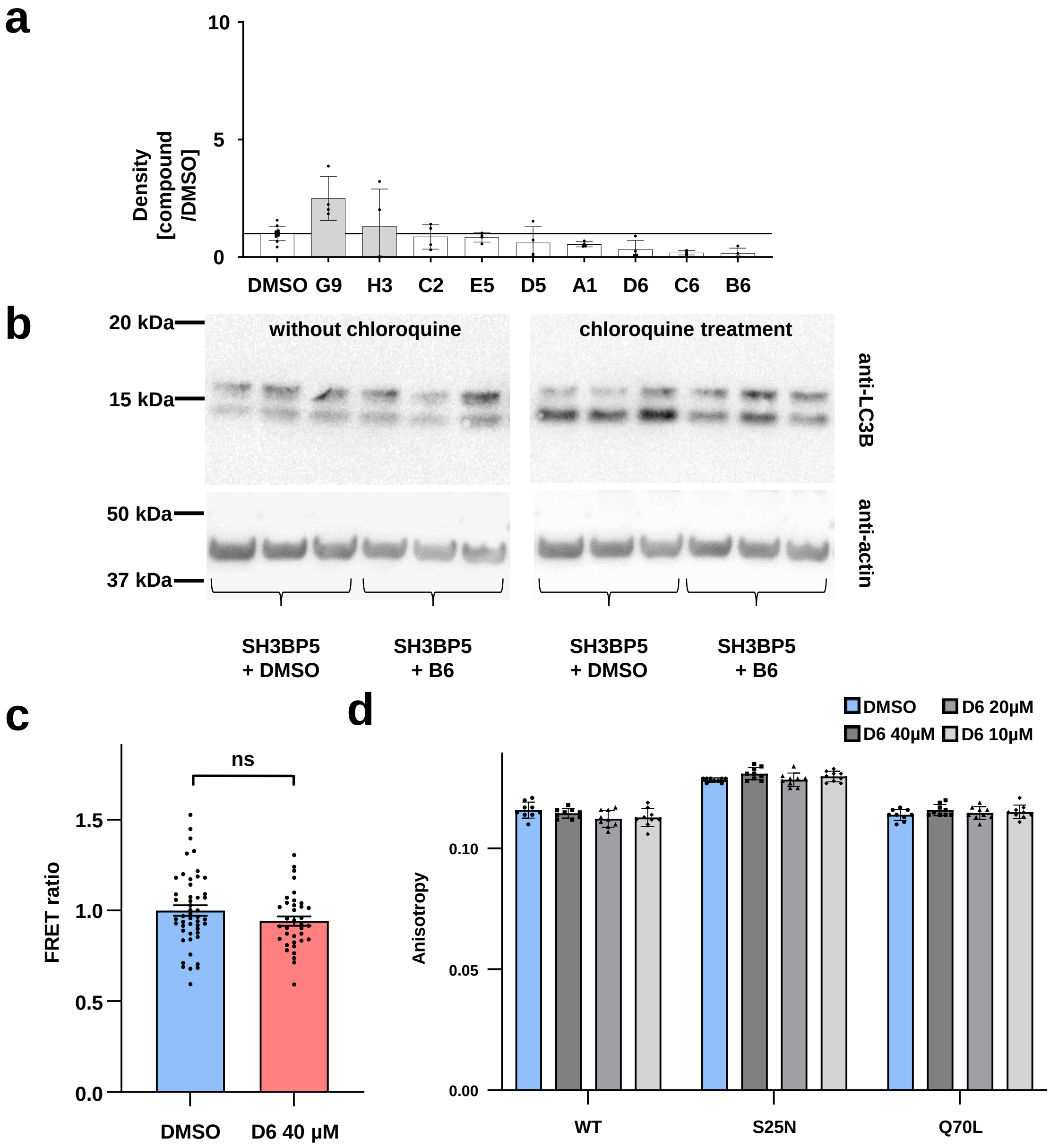

Supplementary Figure 3. Low-dose dataset and chloroquine treatment reveals decreased autophagic flux.

(a) Quantification from immunoblot of supernatant using 9 compounds effective at 20 µM in the primary screen at lower concentration of 5 µM. Two compounds with excessive response were censored (grey).

(b) Immunoblot of supernatant from HEK293T expressing secretory GFP after transfection with SH3BP5 is shown comparing compound B6 with DMSO after chloroquine or control treatment. The intensity of the lower band decreases more strongly after chloroquine, suggesting an inhibition of autophagic flux.

(c) Quantitative analysis of FRET efficiency (FRET ratio) is shown as readout of RAB11A-GTP loading after application of Rab11-inhibitor-D6 at 40 µM for 16 h compared to vehicle does not prevent GTP-loading (mean +/- SE, n = 49 cells for vehicle and 36 cells for Rab11-inhibitor-D6 condition, $p>0.05$).

(d) Quantitative anisotropy-based FRET analysis of RAB11A and C-terminal FIP3 domain interaction i.e. reduction of steady state anisotropy due to the different orientation between the donor and acceptor transition dipole moments in response to the interaction between Rab11 and FIP3. Exposure to Rab11-inhibitor-D6 for 24 h does not increase anisotropy (mean +/- SE, n = 9 per condition, $p>0.05$ for all conditions). S25 is a dominant negative, Q70L a constitutively active variant of RAB11.

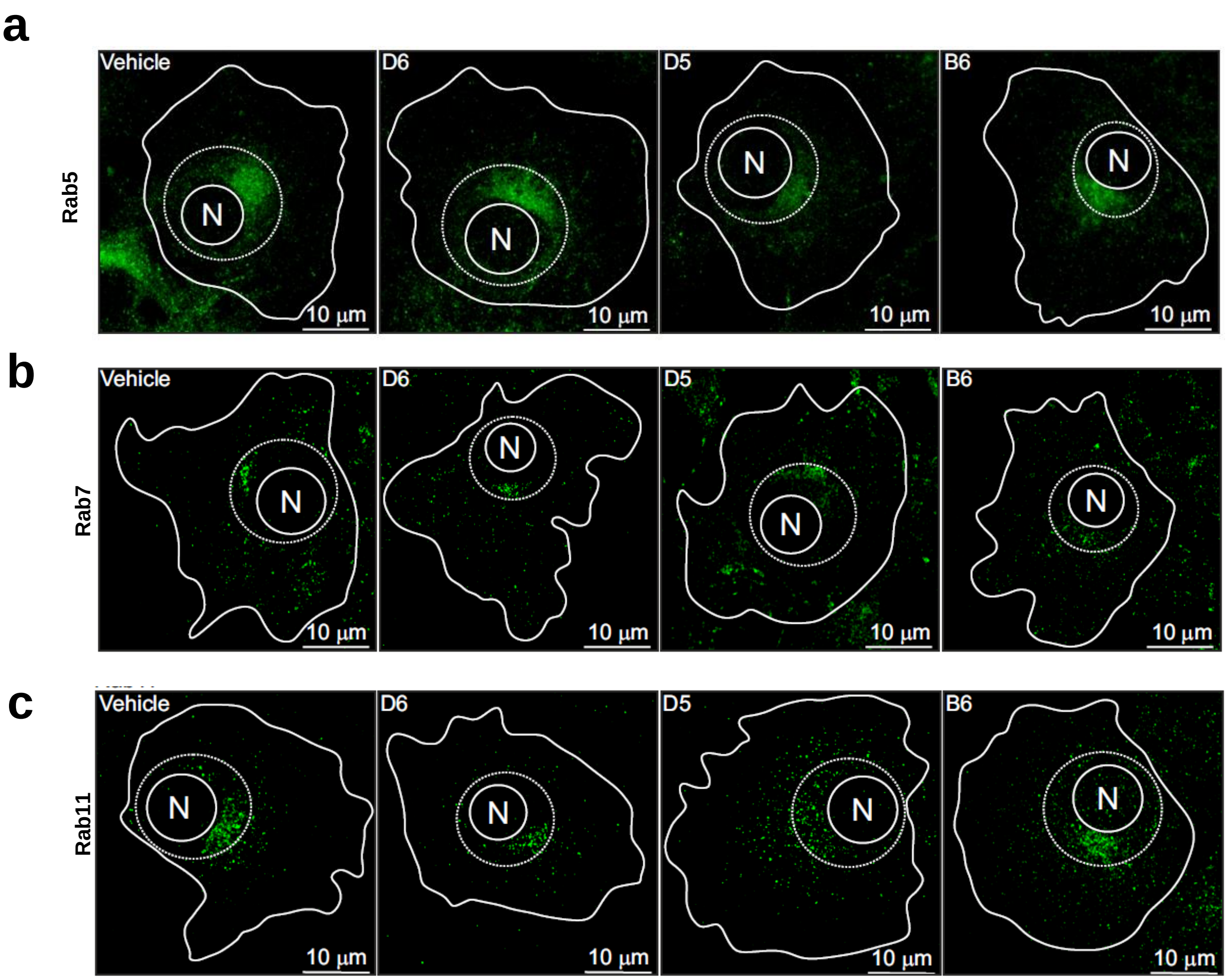

Supplementary Figure 4. Representative images of Rab5/Rab7/Rab11 after treatment with Rab11 inhibitors D6, D5 and B6.

(a-c). Representative images of Rab proteins in a control condition (vehicle) and after treatment with D6, D5, and B6 inhibitor compounds. Cell boundary is highlighted by a white solid line. Nucleus is highlighted by a white solid line and indicated by letter 'N'. The perinuclear region is surrounded in a white dashed line, while the cell periphery is the remaining part of the cell. Images are shownin endogenous Rab5 (a), Rab7 (b), or Rab11 (c).

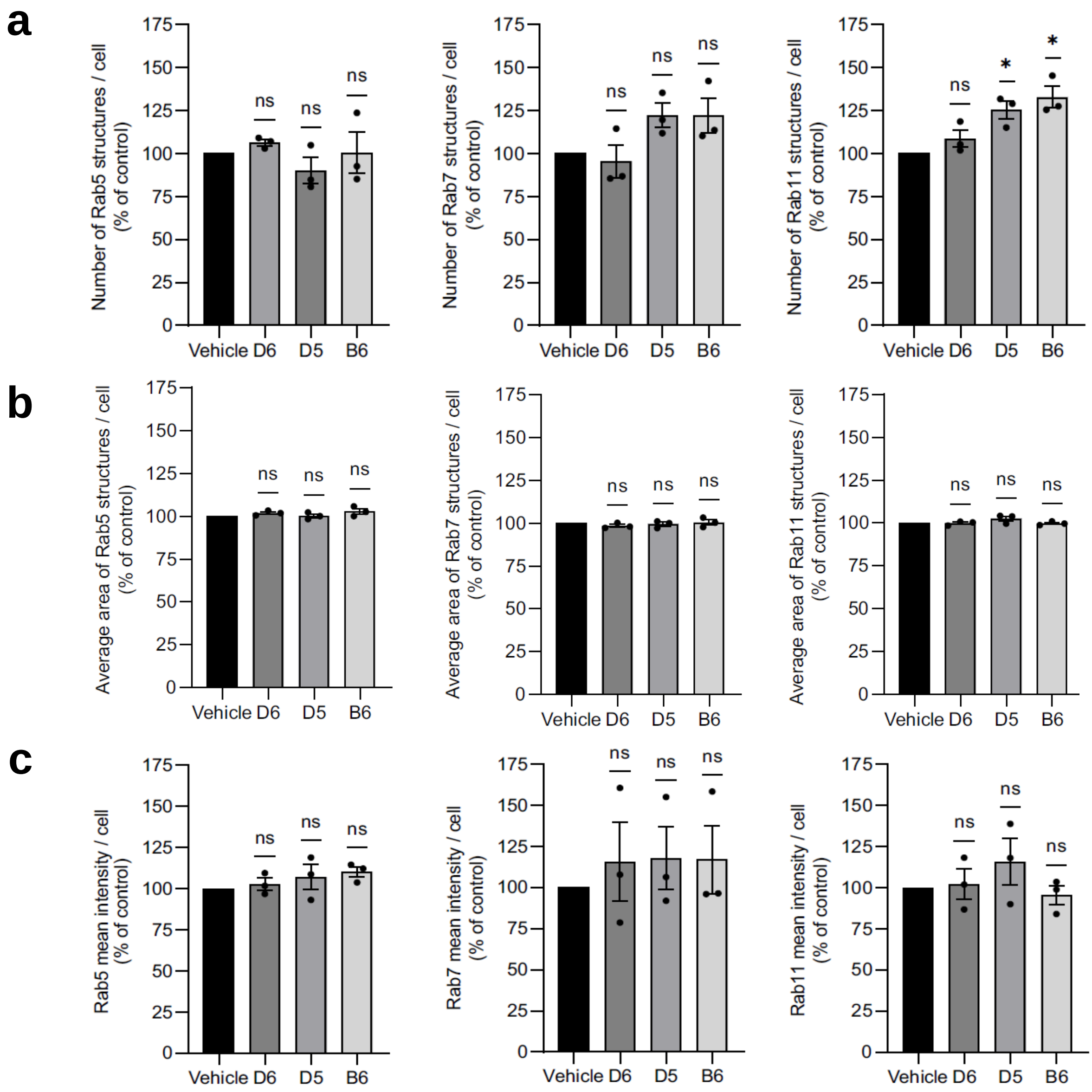

Supplementary Figure 5. Quantification of Rab5/Rab7/Rab11 features shows that D5 and B6 compounds increase the number of Rab11-positive structures.

(**a-c**) Rab5 (left panel), Rab7 (middle panel) and Rab11(right panel) constructs per single cell are shown after treatment with either DMSO (vehicle), D6, D5, or B6 inhibitor compounds. Quantified are vesicle numbers (a), size (b), and fluorescence intensity (c) of the signal from the respective Rab proteins. Error bars represent mean +- S.E.M. n.s. not significant, * $p<0.05$ (one sample Student's t-test). N = 3 independent experiments.

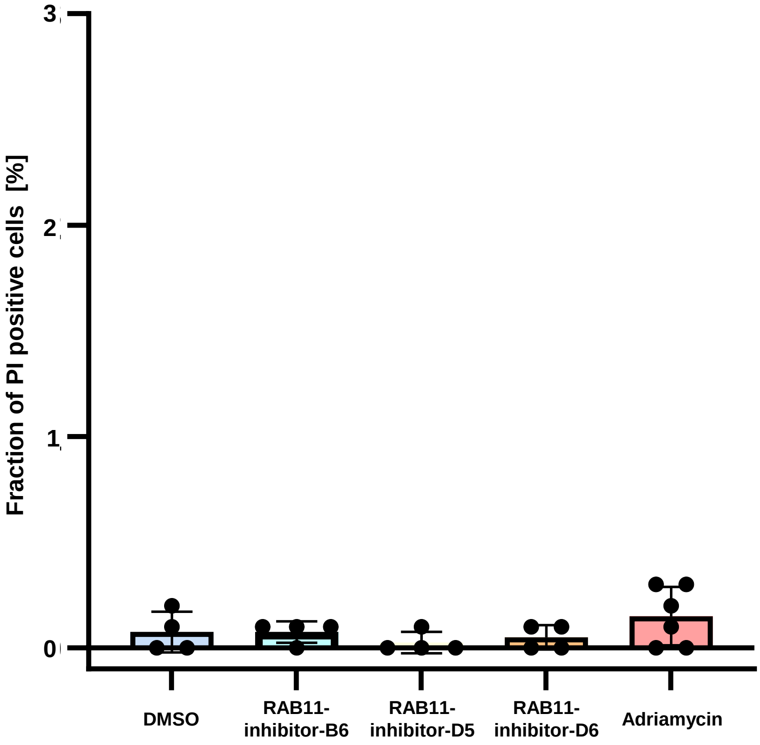

Supplementary Figure 6. Near total absence of exclusively propidium-positive podocytes upon treatment.

Immortalized podocytes were exposed to the respective compounds for 24 hours preceding Annexin V/propidium iodide exposure and flow cytometry. The graph shows the fraction of cells that are positive for propidium iodide but negative for Annexin V (suggesting necrosis) for the indicated conditions. The fraction of exclusively PI-positive cells was <0.2 % for all conditions ($n = 4$, $p>0.05$ for all conditions).

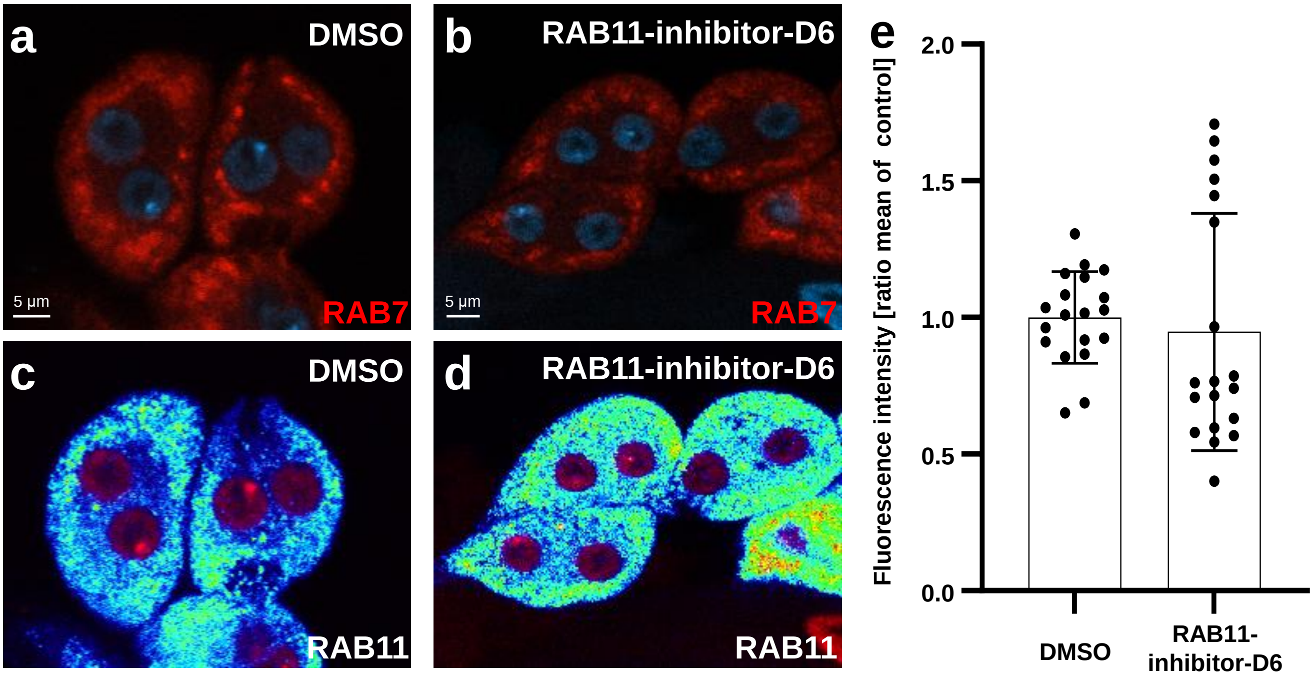

Supplementary Figure 7. Additional stainings of nephrocytes after RAB11-inhibitor-D6 feeding.

(a-b) Confocal images of nephrocytes exposed to RAB11-inhibitor-D6 (b) or DMSO (a) show no overt difference regarding the staining pattern of Rab7.

(c-d) Confocal images of nephrocytes after drug feeding (d) or control (c) are stained for RAB11. Even animals with higher intensity (b) lack a clear vesicular pattern.

(e) Quantification of basal intensity of RAB11 in nephrocytes with/without RAB11-inhibitor-D6 indicates identical intensity on average.

ID	structure	Matrix Plate Position	smiles
Z29251764	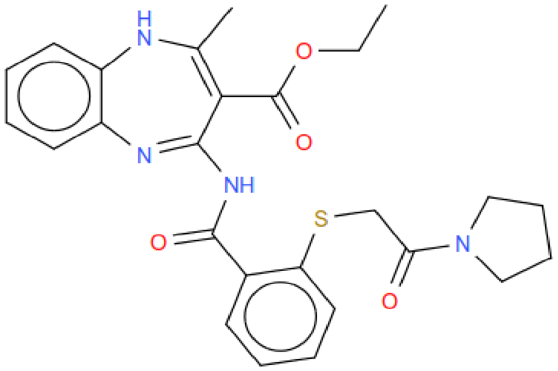	A01	CCOC(=O)C1=C(C)Nc2ccccc2N=C1NC(=O)c1ccccc1SCC(=O)N1CCCC1
Z98997586	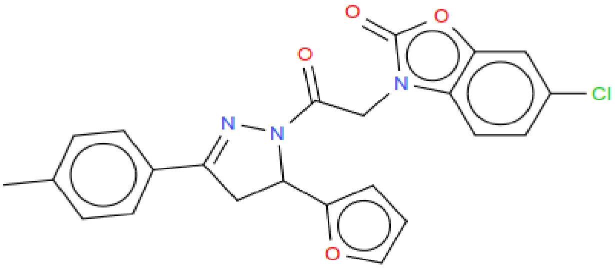	A02	Cc1ccc(C2=NN(C(=O)Cn3c(=O)oc4cc(Cl)ccc43)C(c3ccco3)C2)cc1
Z56977087	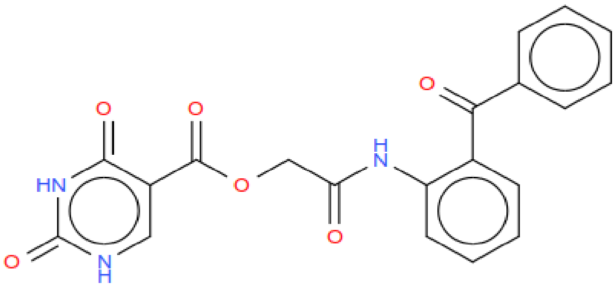	A03	O=C(COC(=O)c1c[nH]c(=O)[nH]c1=O)Nc1ccccc1C(=O)c1ccccc1
Z104366186	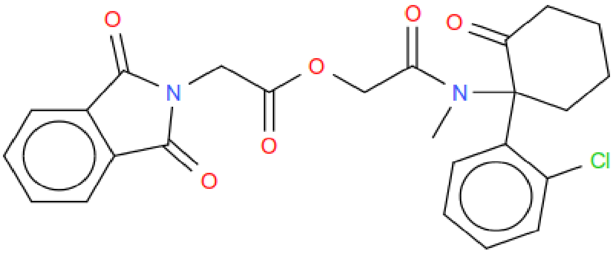	A04	CN(C(=O)COC(=O)CN1C(=O)c2ccccc2C1=O)C1(c2ccccc2Cl)CCCCC1=O
Z227452808	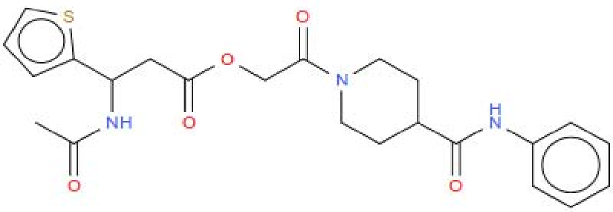	A05	CC(=O)NC(CC(=O)OCC(=O)N1CCC(C(=O)Nc2ccccc2)CC1)c1cccs1

Suppl. Table 1. Compounds evaluated in the screen (part 1)

Z2184465219	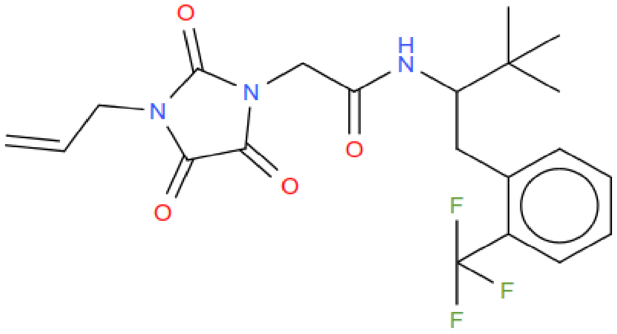	A06	C=CCN1C(=O)C(=O)N(CC(=O)NC(Cc2ccccc2C(F)(F)F)C(C)(C)C)C1=O
Z17411026	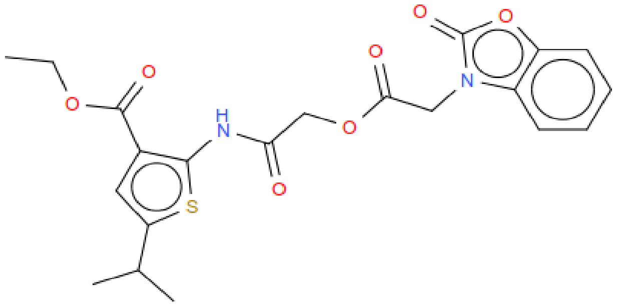	A07	CCOC(=O)c1cc(C(C)C)sc1NC(=O)COC(=O)Cn1c(=O)oc2ccccc21
Z55429060	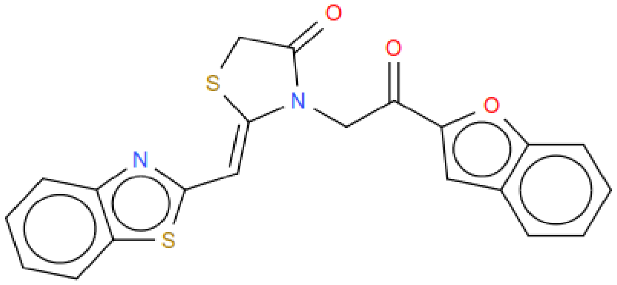	A08	O=C(CN1C(=O)CSC1=Cc1nc2ccccc2s1)c1cc2ccccc2o1
Z56909115	Chiral<br>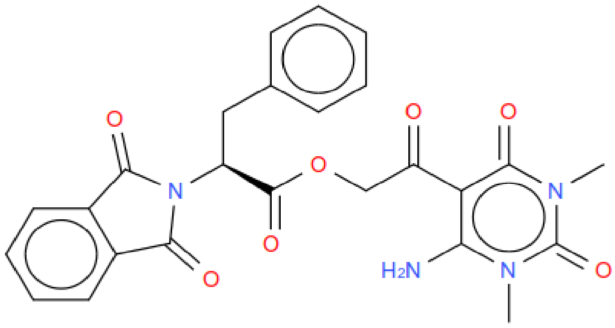	A09	Cn1c(N)c(C(=O)COC(=O)[C@H](Cc2ccccc2)N2C(=O)c3ccccc3C2=O)c(=O)n(C)c1=O
Z223944014	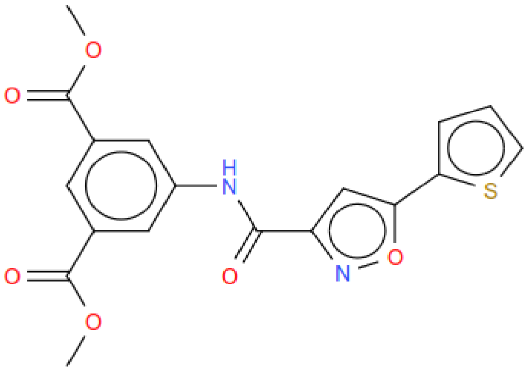	A10	COC(=O)c1cc(NC(=O)c2cc(-c3cccs3)on2)cc(C(=O)OC)c1

Suppl. Table 1. Compounds evaluated in the screen (part 2)

Z223650138	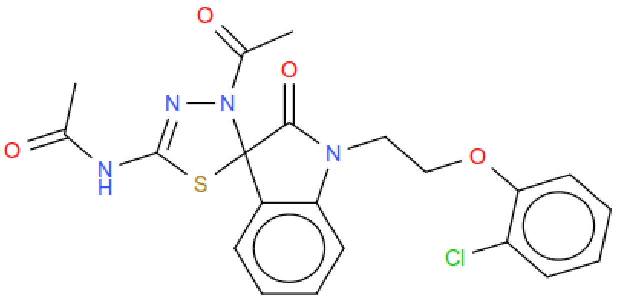	A11	CC(=O)NC1=NN(C(C)=O)C2(S1)C(=O)N(CCOc1ccccc1Cl)c1ccccc12
Z29385046	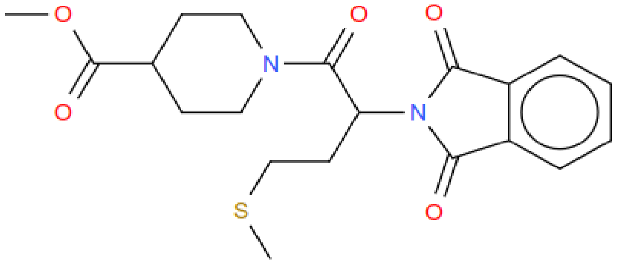	A12	COC(=O)C1CCN(C(=O)C(CCSC)N2C(=O)c3ccccc3C2=O)CC1
Z24758175	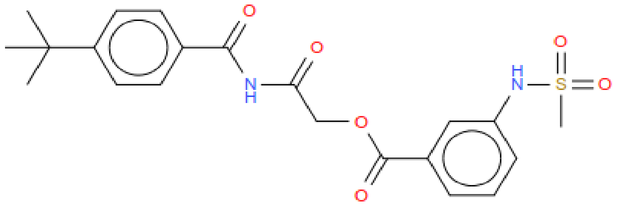	B01	CC(C)(C)c1ccc(C(=O)NC(=O)COC(=O)c2cccc(NS(C)(=O)=O)c2)cc1
Z87951730	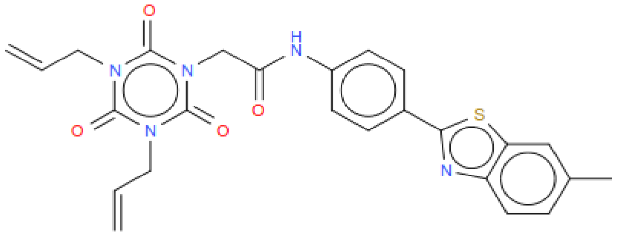	B02	C=CCn1c(=O)n(CC=C)c(=O)n(CC(=O)Nc2ccc(-c3nc4ccc(C)cc4s3)cc2)c1=O
Z278082790	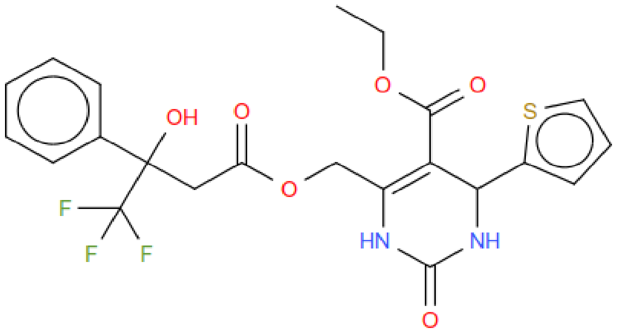	B03	CCOC(=O)C1=C(COC(=O)CC(O)(c2ccccc2)C(F)(F)F)NC(=O)NC1c1cccs1

Suppl. Table 1. Compounds evaluated in the screen (part 3)

Z425292842	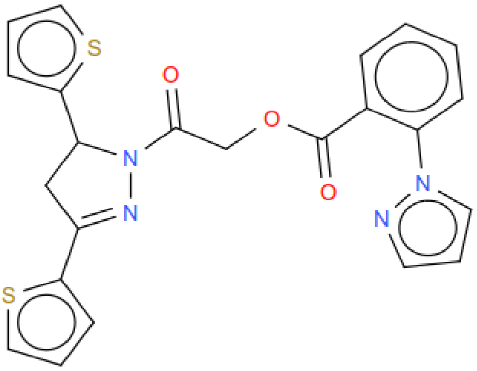	B04	O=C(OCC(=O)N1N=C(c2cccs2)CC1c1cccs1)c1ccccc1-n1cccn1
Z437222772	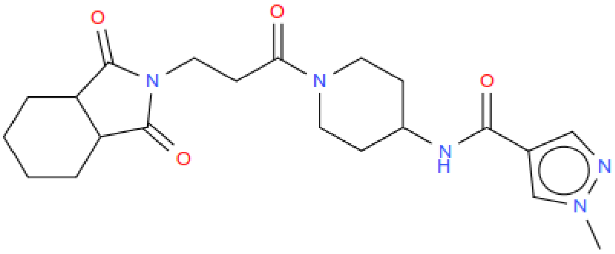	B05	Cn1cc(C(=O)NC2CCN(C(=O)CCN3C(=O)C4CCCCC4C3=O)CC2)cn1
Z17937228	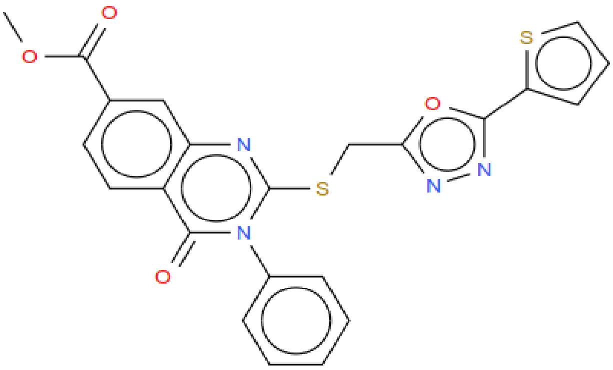	B06	COC(=O)c1ccc2c(=O)n(-c3ccccc3)c(SCc3nnc(-c4cccs4)o3)nc2c1
Z194567502	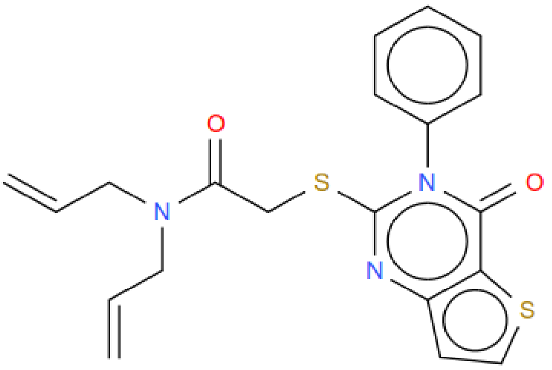	B07	C=CCN(CC=C)C(=O)CSc1nc2ccsc2c(=O)n1-c1ccccc1
Z85929816	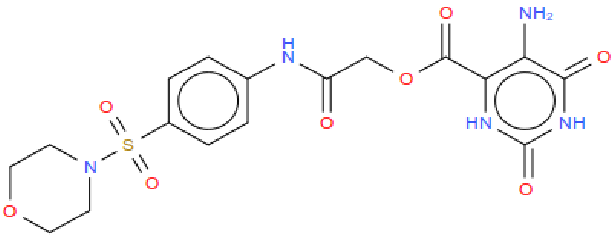	B08	Nc1c(C(=O)OCC(=O)Nc2ccc(S(=O)(=O)N3CCOCC3)cc2)[nH]c(=O)[nH]c1=O

Suppl. Table 1. Compounds evaluated in the screen (part 4)

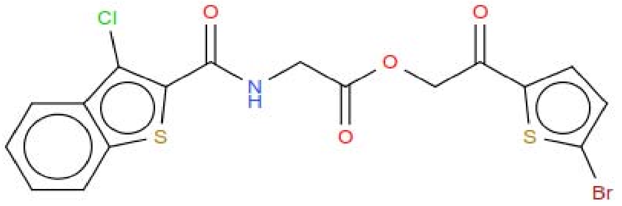

Z13950842 B09 O=C(CNC(=O)c1sc2ccccc2c1Cl)OCC(=O)c1ccc(Br)s1

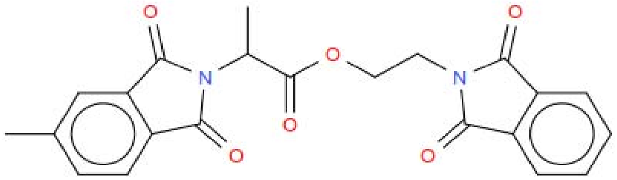

Z82068234 B10 Cc1ccc2c(c1)C(=O)N(C(C)C(=O)OCCN1C(=O)c3ccccc3C1=O)C2=O

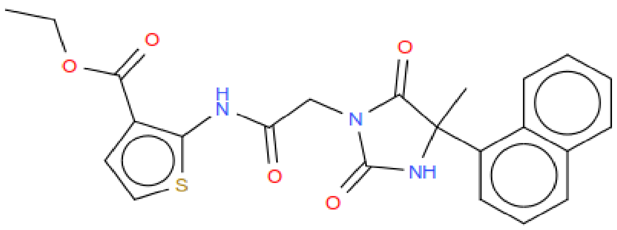

Z199690178 B11 CCOC(=O)c1ccsc1NC(=O)CN1C(=O)NC(C)(c2cccc3ccccc23)C1=O

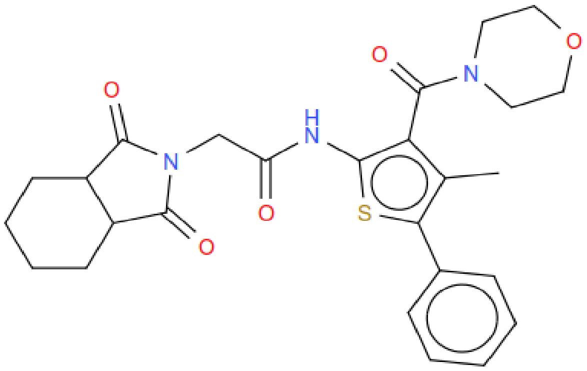

Z28006583 B12 Cc1c(-c2ccccc2)sc(NC(=O)CN2C(=O)C3CCCCC3C2=O)c1C(=O)N1CCOCC1

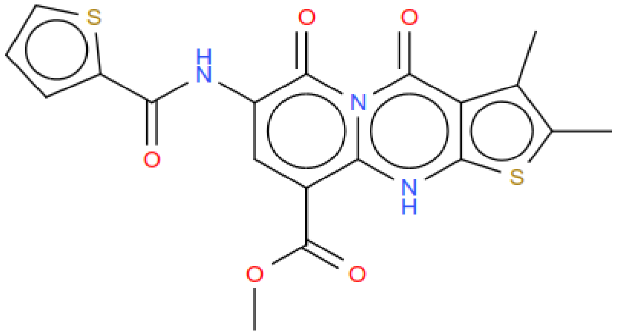

Z90665009 C01 COC(=O)c1cc(NC(=O)c2cccs2)c(=O)n2c(=O)c3c(C)c(C)sc3[nH]c12

Suppl. Table 1. Compounds evaluated in the screen (part 5)

Z18977093	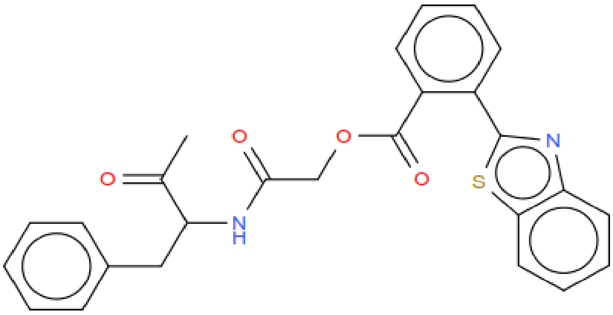	C02	CC(=O)C(Cc1ccccc1)NC(=O)CO C(=O)c1ccccc1-c1nc2ccccc2s1
Z92671387	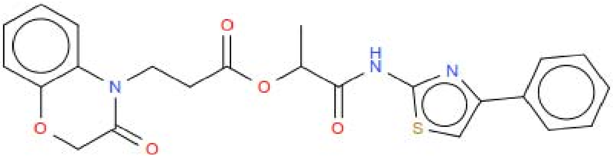	C03	CC(OC(=O)CCN1C(=O)COc2cc ccc21)C(=O)Nc1nc(- c2ccccc2)cs1
Z24192421	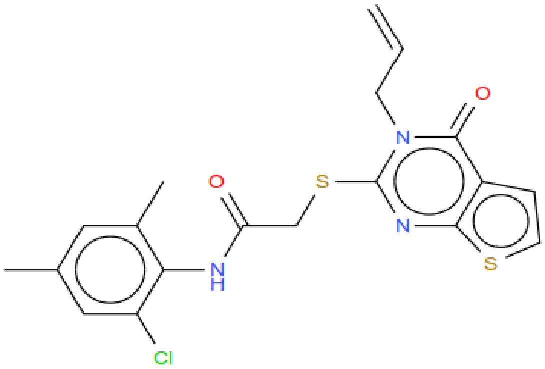	C04	C=CCn1c(SCC(=O)Nc2c(C)cc(C)cc2Cl)nc2sccc2c1=O
Z17112883	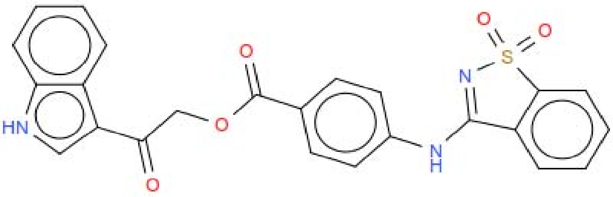	C05	O=C(OCC(=O)c1c[nH]c2ccccc1 2)c1ccc(NC2=NS(=O)(=O)c3ccc cc32)cc1
Z1290173781	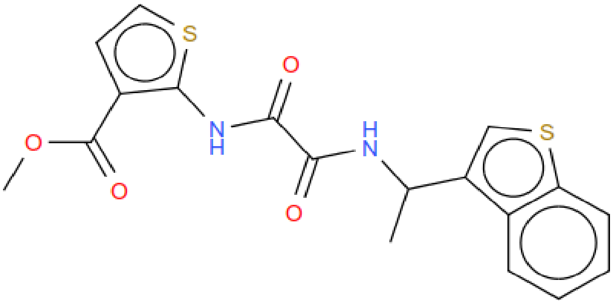	C06	COC(=O)c1ccsc1NC(=O)C(=O) NC(C)c1csc2ccccc12

Suppl. Table 1. Compounds evaluated in the screen (part 6)

Z88160390	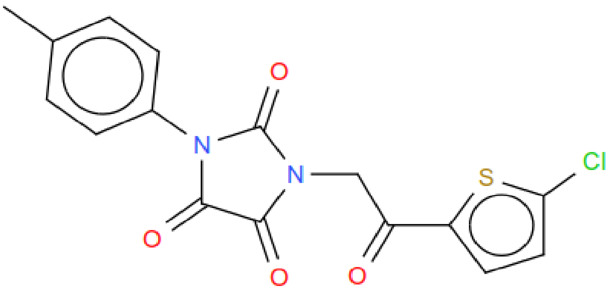	C07	Cc1ccc(N2C(=O)C(=O)N(CC(=O)c3ccc(Cl)s3)C2=O)cc1
Z85920426	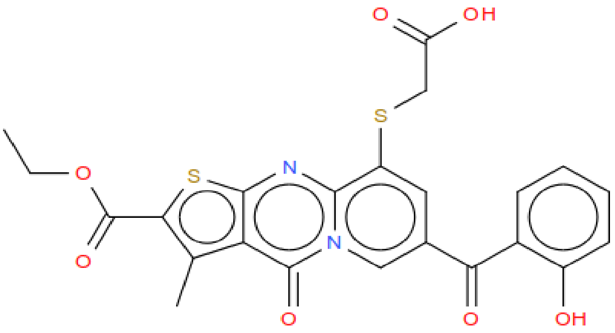	C08	CCOC(=O)c1sc2nc3c(SCC(=O)O)cc(C(=O)c4ccccc4O)cn3c(=O)c2c1C
Z56962236	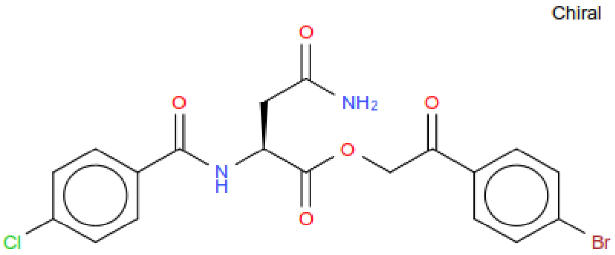	C09	NC(=O)C[C@H](NC(=O)c1ccc(Cl)cc1)C(=O)OCC(=O)c1ccc(Br)cc1
Z951171638	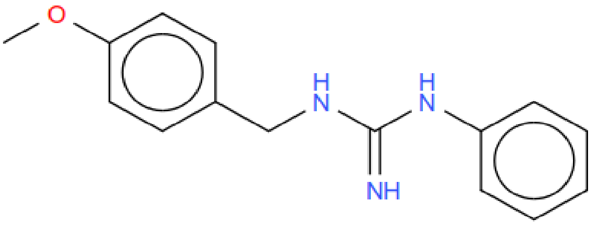	C10	COc1ccc(CNC(=N)Nc2ccccc2)cc1
Z66391696	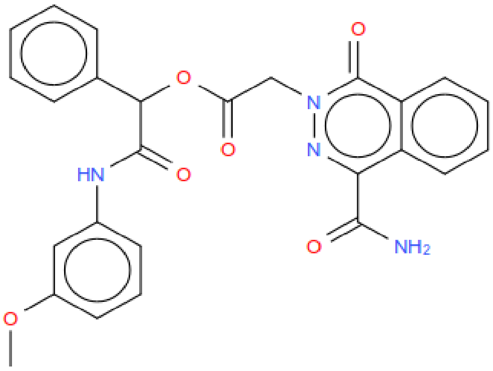	C11	COc1cccc(NC(=O)C(OC(=O)Cn2nc(C(N)=O)c3ccccc3c2=O)c2ccccc2)c1

Suppl. Table 1. Compounds evaluated in the screen (part 7)

Z808674308	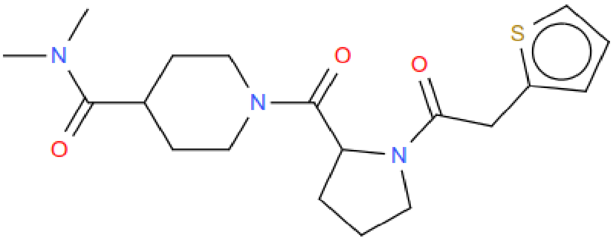	C12	CN(C)C(=O)C1CCN(C(=O)C2CCCN2C(=O)Cc2cccs2)CC1
Z27933284	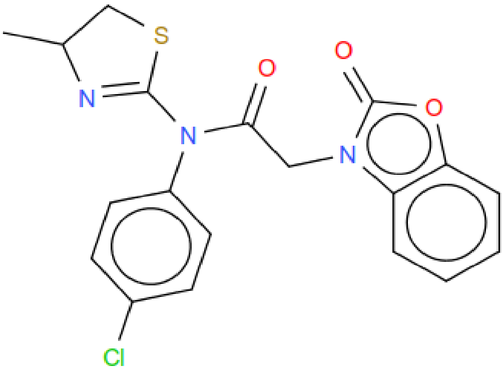	D01	CC1CSC(N(C(=O)Cn2c(=O)oc3ccccc32)c2ccc(Cl)cc2)=N1
Z14096582	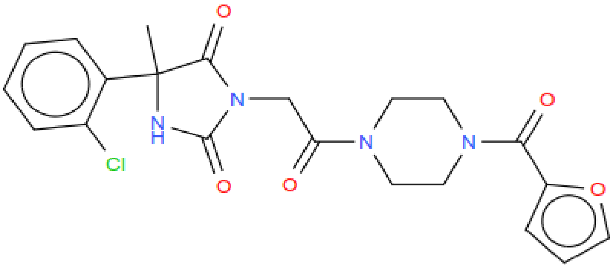	D02	CC1(c2ccccc2Cl)NC(=O)N(CC(=O)N2CCN(C(=O)c3ccco3)CC2)C1=O
Z90072083	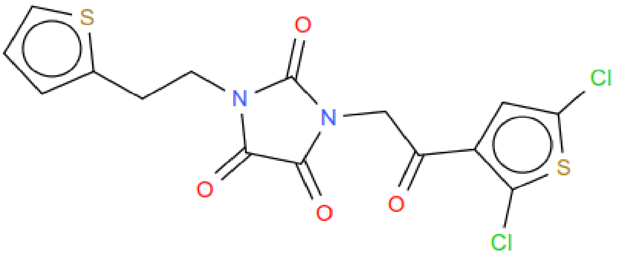	D03	O=C(CN1C(=O)C(=O)N(CCc2cccs2)C1=O)c1cc(Cl)sc1Cl
Z88593129	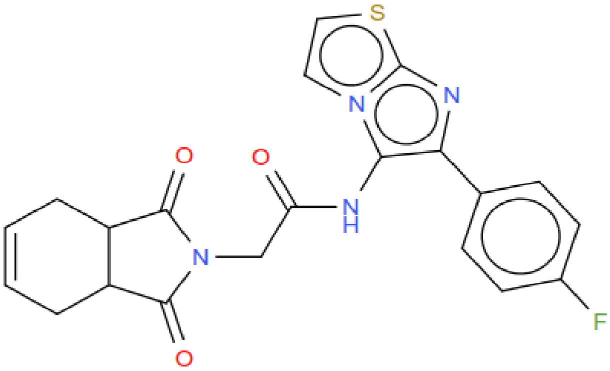	D04	O=C(CN1C(=O)C2CC=CCC2C1=O)Nc1c(-c2ccc(F)cc2)nc2sccn12

Suppl. Table 1. Compounds evaluated in the screen (part 8)

Z66498322	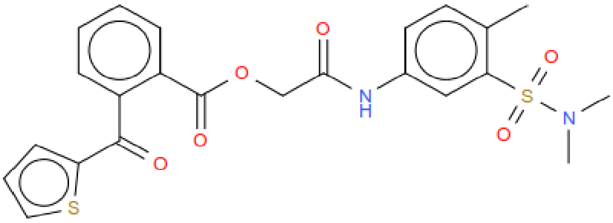	D05	Cc1ccc(NC(=O)COC(=O)c2cccc<br>c2C(=O)c2cccs2)cc1S(=O)(=O)<br>N(C)C
Z57011850	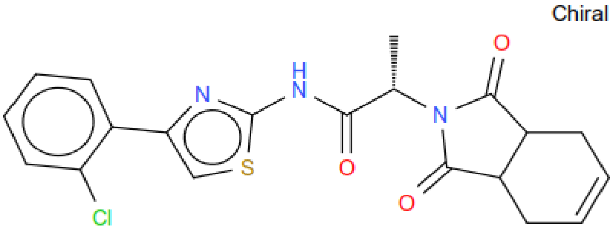	D06	C[C@@H](C(=O)Nc1nc(-<br>c2ccccc2Cl)cs1)N1C(=O)C2CC=<br>CCC2C1=O
Z74266645	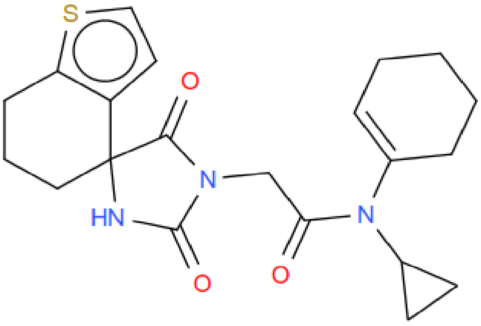	D07	O=C1NC2(CCCc3sccc32)C(=O)<br>N1CC(=O)N(C1=CCCCC1)C1CC<br>1
Z81731470	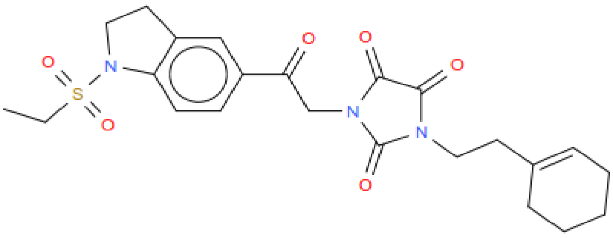	D08	CCS(=O)(=O)N1CCc2cc(C(=O)C<br>N3C(=O)C(=O)N(CCC4=CCCCC<br>4)C3=O)ccc21
Z74278672	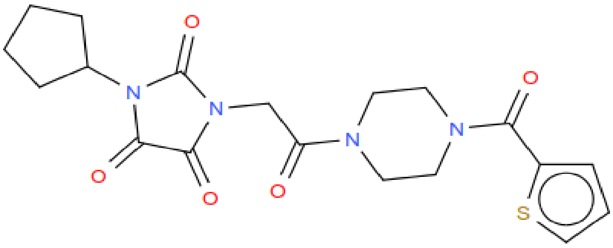	D09	O=C(CN1C(=O)C(=O)N(C2CCC<br>C2)C1=O)N1CCN(C(=O)c2cccs2<br>)CC1

Suppl. Table 1. Compounds evaluated in the screen (part 9)

Z1344864350	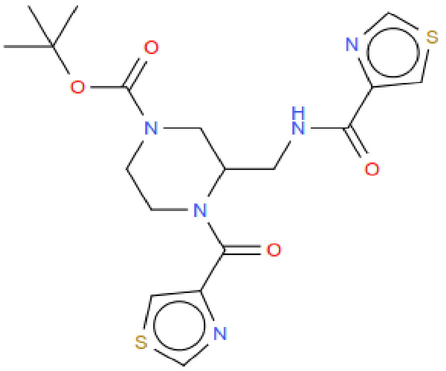	D10	CC(C)(C)OC(=O)N1CCN(C(=O)c2cscn2)C(CNC(=O)c2cscn2)C1
Z18471792	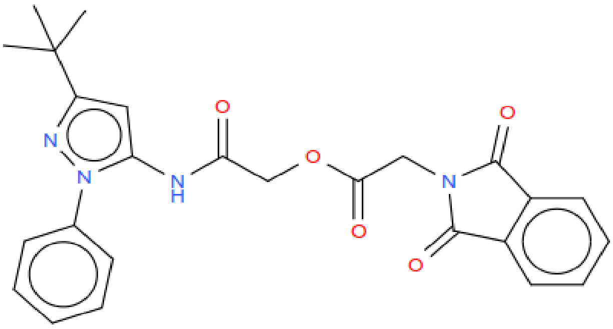	D11	CC(C)(C)c1cc(NC(=O)COC(=O)CN2C(=O)c3ccccc3C2=O)n(-c2ccccc2)n1
Z3214698931	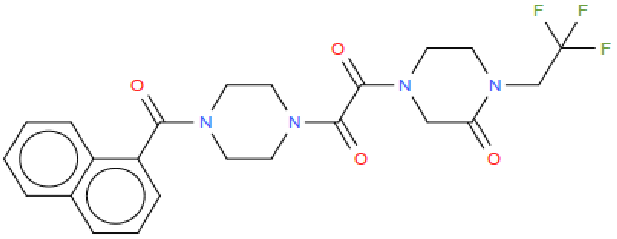	D12	O=C1CN(C(=O)C(=O)N2CCN(C(=O)c3cccc4ccccc34)CC2)CCN1CC(F)(F)F
Z45210395	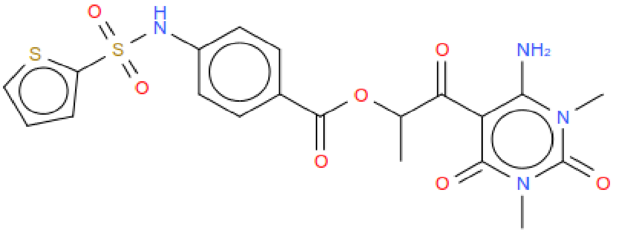	E01	CC(OC(=O)c1ccc(NS(=O)(=O)c2cccs2)cc1)C(=O)c1c(N)n(C)c(=O)n(C)c1=O
Z16298341	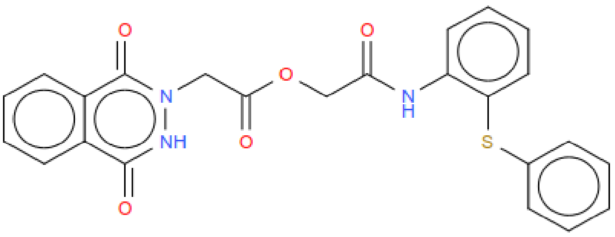	E02	O=C(COC(=O)Cn1[nH]c(=O)c2ccccc2c1=O)Nc1ccccc1Sc1ccccc1

Suppl. Table 1. Compounds evaluated in the screen (part 10)

Z238335716	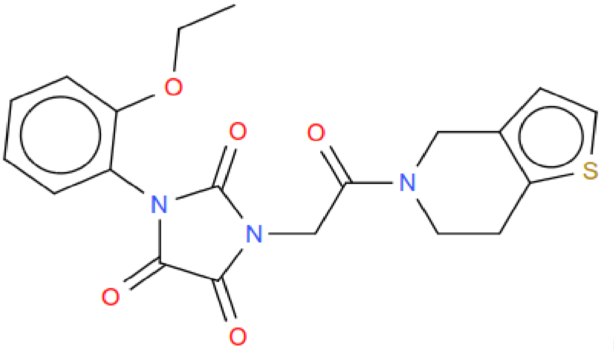	E03	CCOc1ccccc1N1C(=O)C(=O)N(CC(=O)N2CCc3sccc3C2)C1=O
Z98559595	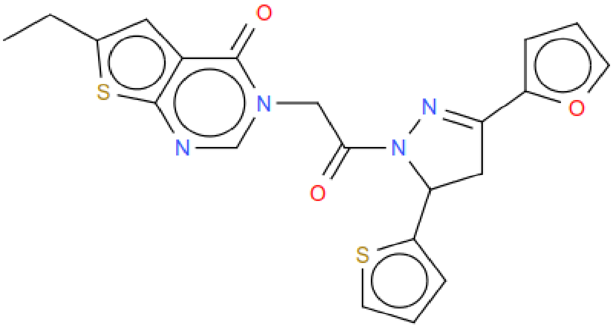	E04	CCc1cc2c(=O)n(CC(=O)N3N=C(c4ccco4)CC3c3cccs3)cnc2s1
Z1665447790	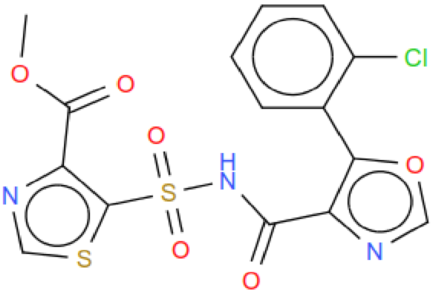	E05	COC(=O)c1ncsc1S(=O)(=O)NC(=O)c1ncoc1-c1ccccc1Cl
Z17114435	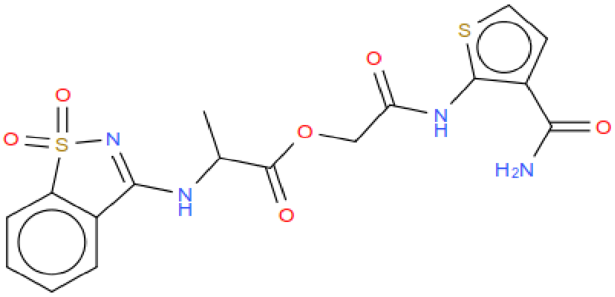	E06	CC(NC1=NS(=O)(=O)c2ccccc21)C(=O)OCC(=O)Nc1sccc1C(N)=O
Z57035638	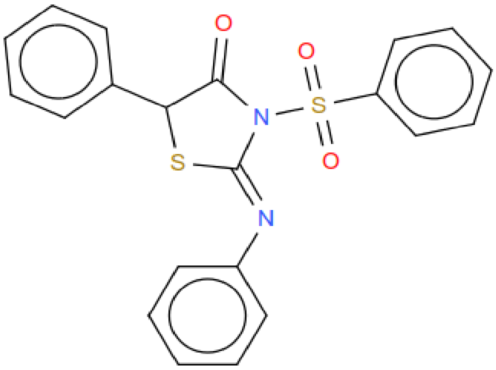	E07	O=C1C(c2ccccc2)SC(=Nc2ccccc2)N1S(=O)(=O)c1ccccc1

Suppl. Table 1. Compounds evaluated in the screen (part 11)

Z104369216	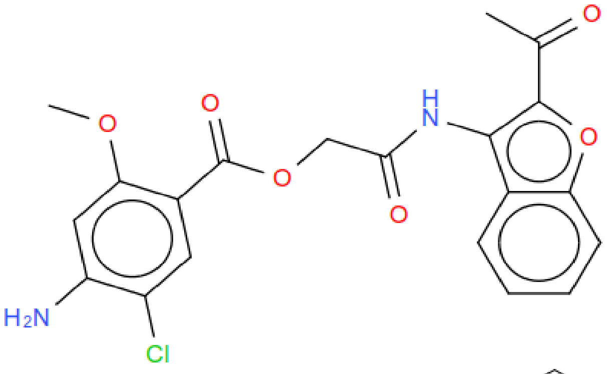	E08	COc1cc(N)c(Cl)cc1C(=O)OCC(=<br>O)Nc1c(C(C)=O)oc2ccccc12
Z18455715	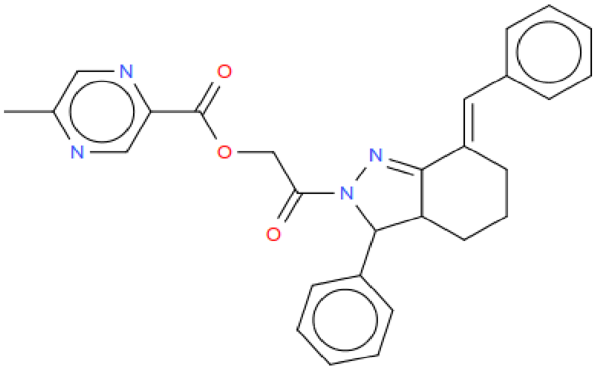	E09	Cc1cnc(C(=O)OCC(=O)N2N=C3<br>C(=Cc4ccccc4)CCCC3C2c2cccc<br>c2)cn1
Z57052351	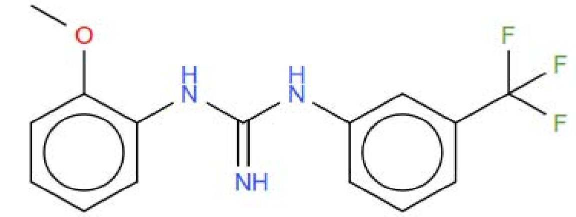	E10	COc1ccccc1NC(=N)Nc1cccc(C(<br>F)(F)F)c1
Z47037662	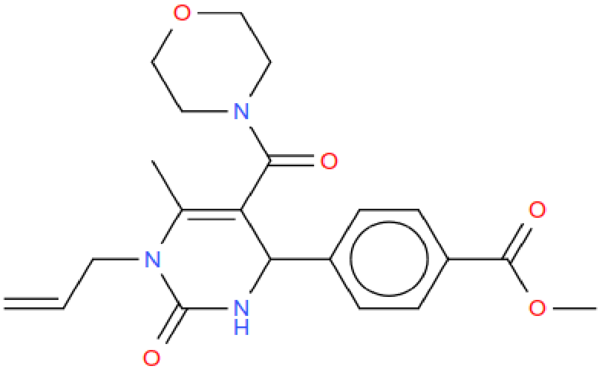	E11	C=CCN1C(=O)NC(c2ccc(C(=O)<br>OC)cc2)C(C(=O)N2CCOCC2)=C<br>1C
Z1190458970	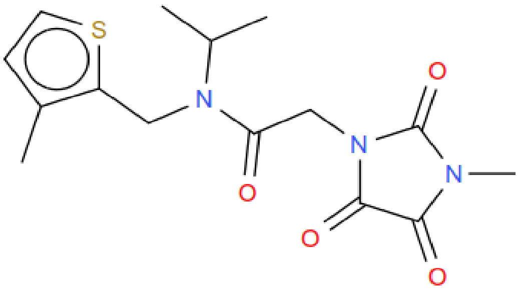	E12	Cc1ccsc1CN(C(=O)CN1C(=O)C(<br>=O)N(C)C1=O)C(C)C

Suppl. Table 1. Compounds evaluated in the screen (part 12)

Z79580415	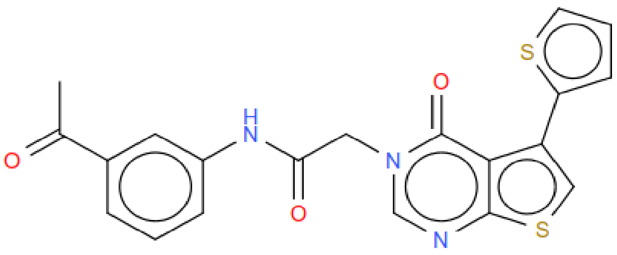	F01	CC(=O)c1cccc(NC(=O)Cn2cnc3scc(-c4cccs4)c3c2=O)c1
Z223060614	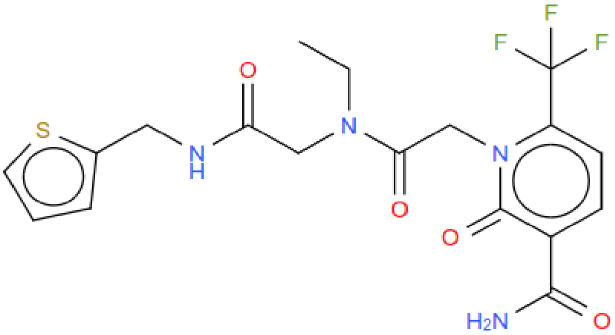	F02	CCN(CC(=O)NCc1cccs1)C(=O)Cn1c(C(F)(F)F)ccc(C(N)=O)c1=O
Z25937062	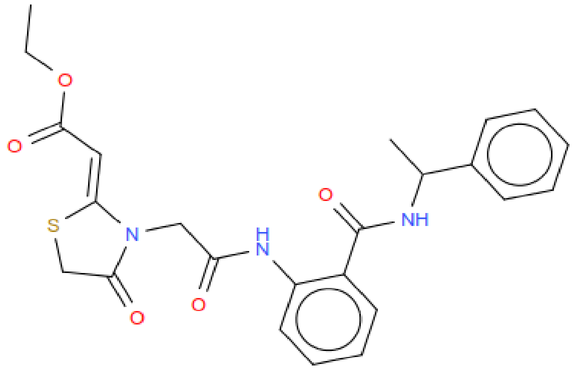	F03	CCOC(=O)C=C1SCC(=O)N1CC(=O)Nc1ccccc1C(=O)NC(C)c1ccccc1
Z22397994	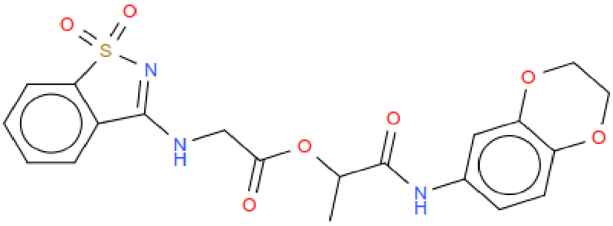	F04	CC(OC(=O)CNC1=NS(=O)(=O)c2ccccc21)C(=O)Nc1ccc2c(c1)OCCO2
Z606163876	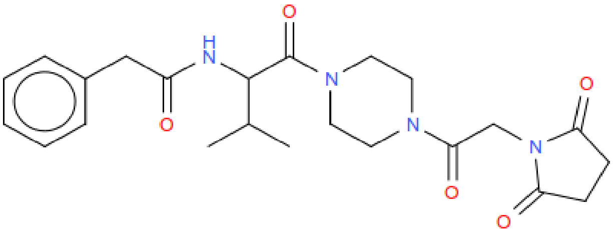	F05	CC(C)C(NC(=O)Cc1ccccc1)C(=O)N1CCN(C(=O)CN2C(=O)CCC2=O)CC1

Suppl. Table 1. Compounds evaluated in the screen (part 13)

Z25790447	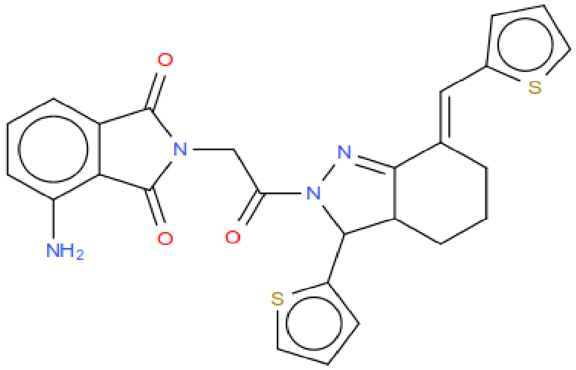	F06	Nc1cccc2c1C(=O)N(CC(=O)N1N=C3C(=Cc4cccs4)CCCC3C1c1cccs1)C2=O
Z14728930	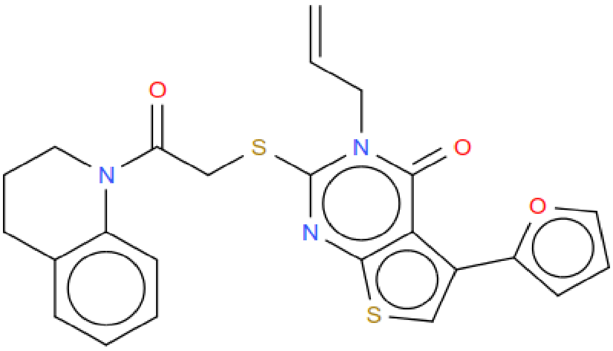	F07	C=CCn1c(SCC(=O)N2CCCc3ccccc32)nc2scc(-c3ccco3)c2c1=O
Z1954757679	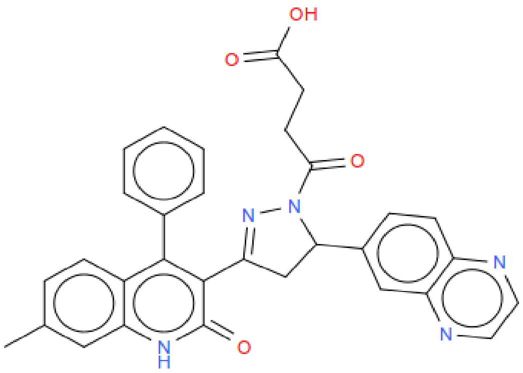	F08	Cc1ccc2c(-c3ccccc3)c(C3=NN(C(=O)CCC(=O)O)C(c4ccc5nccnc5c4)C3)c(=O)[nH]c2c1
Z153345634	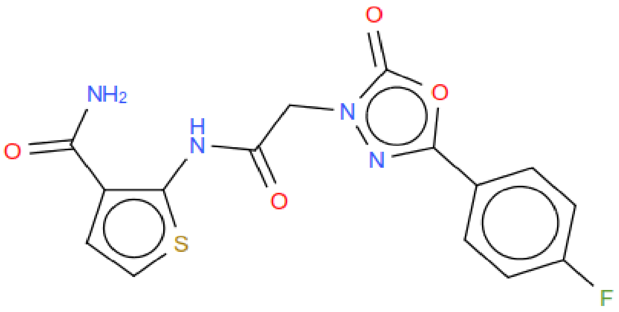	F09	NC(=O)c1ccsc1NC(=O)Cn1nc(-c2ccc(F)cc2)oc1=O
Z17035401	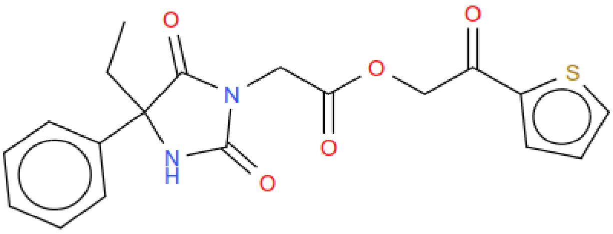	F10	CCC1(c2ccccc2)NC(=O)N(CC(=O)OCC(=O)c2cccs2)C1=O

Suppl. Table 1. Compounds evaluated in the screen (part 14)

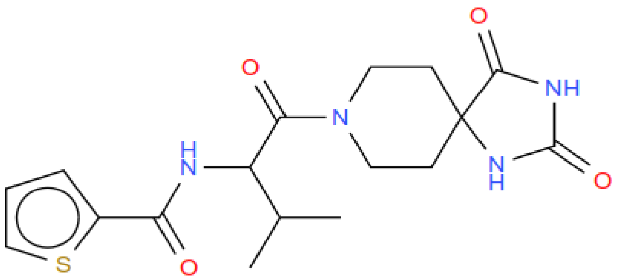

Z225727728 F11 CC(C)C(NC(=O)c1cccs1)C(=O)N1CCC2(CC1)NC(=O)NC2=O

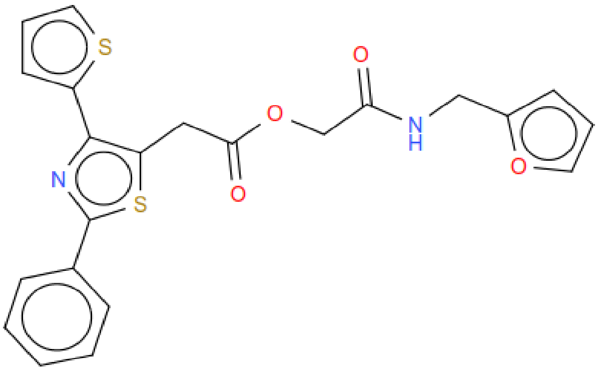

Z13914860 F12 O=C(COC(=O)Cc1sc(-c2ccccc2)nc1-c1cccs1)NCc1ccco1

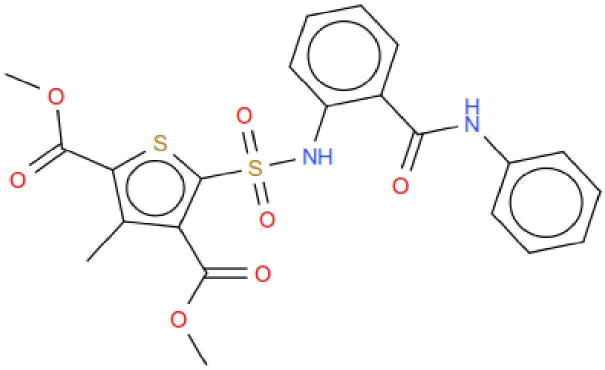

Z45580442 G01 COC(=O)c1sc(S(=O)(=O)Nc2ccccc2C(=O)Nc2ccccc2)c(C(=O)OC)c1C

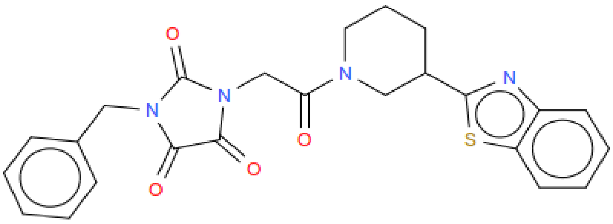

Z97372909 G02 O=C(CN1C(=O)C(=O)N(Cc2ccccc2)C1=O)N1CCCC(c2nc3ccccc3s2)C1

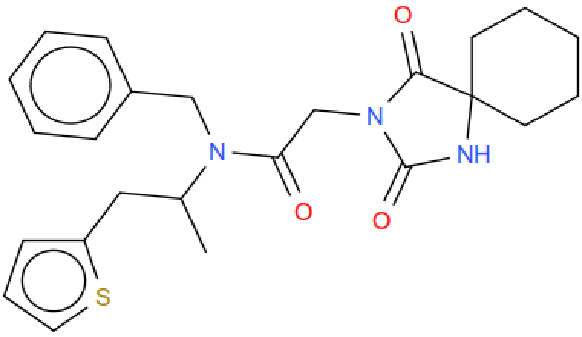

Z29146064 G03 CC(Cc1cccs1)N(Cc1ccccc1)C(=O)CN1C(=O)NC2(CCCCC2)C1=O

Suppl. Table 1. Compounds evaluated in the screen (part 15)

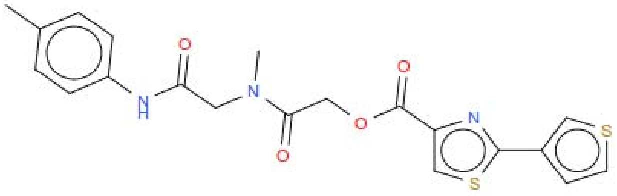

Z194092010 G04 Cc1ccc(NC(=O)CN(C)C(=O)CO C(=O)c2csc(-c3ccsc3)n2)cc1

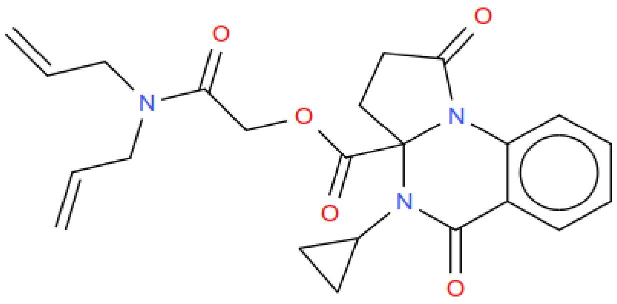

Z92622309 G05 C=CCN(CC=C)C(=O)COC(=O)C 12CCC(=O)N1c1ccccc1C(=O)N2 C1CC1

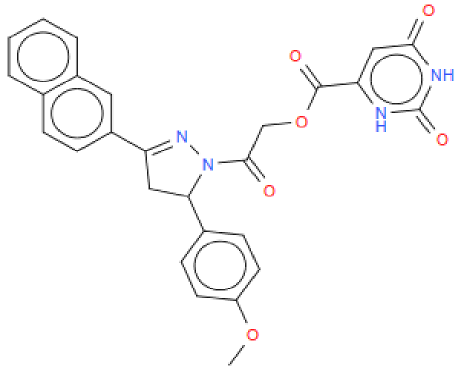

Z57050781 G06 COc1ccc(C2CC(c3ccc4ccccc4c 3)=NN2C(=O)COC(=O)c2cc(=O) [nH]c(=O)[nH]2)cc1

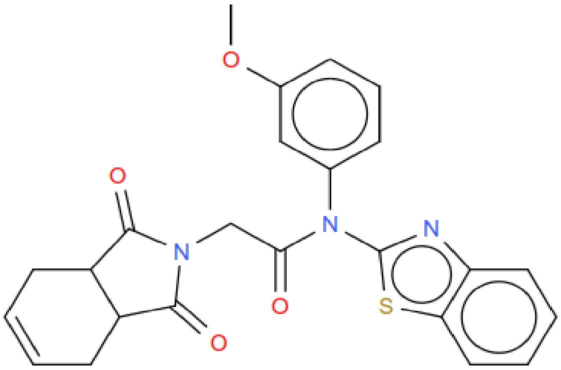

Z27407872 G07 COc1cccc(N(C(=O)CN2C(=O)C3 CC=CCC3C2=O)c2nc3ccccc3s2)c1

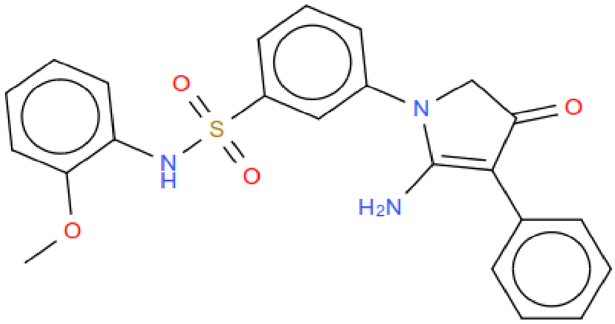

Z56777846 G08 COc1ccccc1NS(=O)(=O)c1cccc(N2CC(=O)C(c3ccccc3)=C2N)c1

Suppl. Table 1. Compounds evaluated in the screen (part 16)

Z17090950	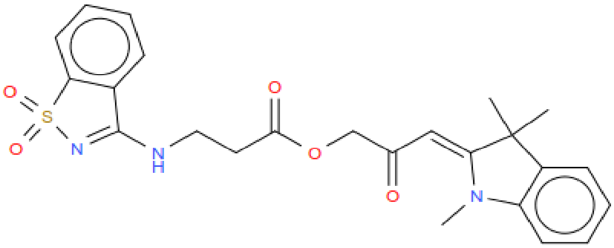	G09	CN1C(=CC(=O)COC(=O)CCNC2=NS(=O)(=O)c3ccccc32)C(C)(C)c2ccccc21
Z167768874	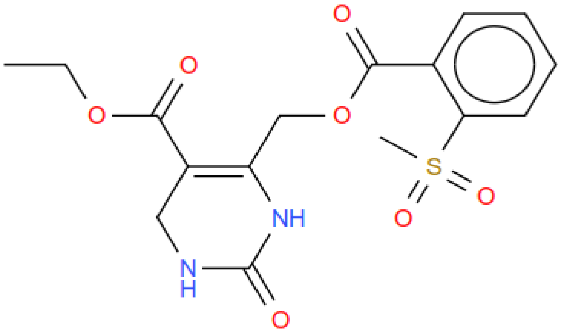	G10	CCOC(=O)C1=C(COC(=O)c2ccccc2S(C)(=O)=O)NC(=O)NC1
Z15583438	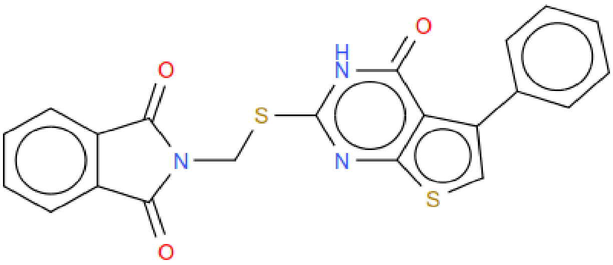	G11	O=C1c2ccccc2C(=O)N1CSc1nc2scc(-c3ccccc3)c2c(=O)[nH]1
Z90277224	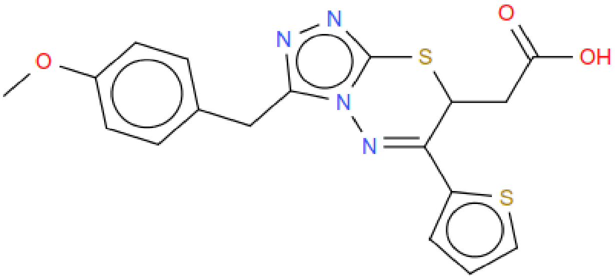	H01	COc1ccc(Cc2nnc3n2N=C(c2cccs2)C(CC(=O)O)S3)cc1
Z109483306	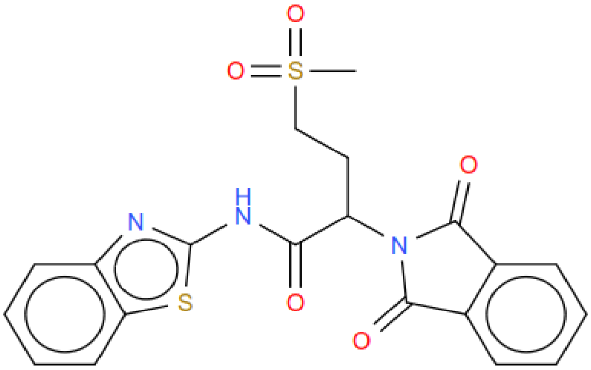	H02	CS(=O)(=O)CCC(C(=O)Nc1nc2ccccc2s1)N1C(=O)c2ccccc2C1=O

Suppl. Table 1. Compounds evaluated in the screen (part 17)

Z29748531	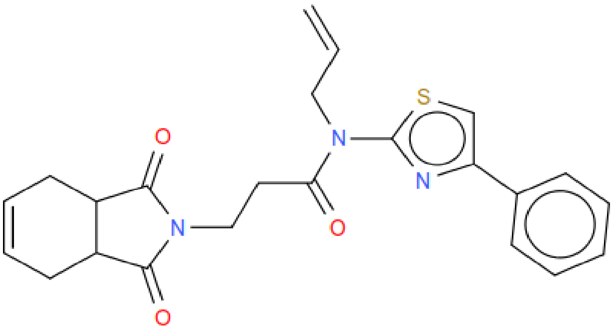	H03	C=CCN(C(=O)CCN1C(=O)C2CC=CCC2C1=O)c1nc(-c2ccccc2)cs1
Z18754653	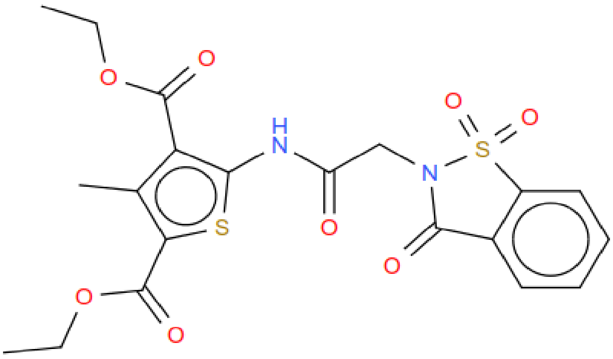	H04	CCOC(=O)c1sc(NC(=O)CN2C(=O)c3ccccc3S2(=O)=O)c(C(=O)OCC)c1C
Z915382786	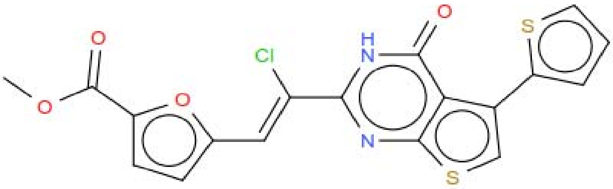	H05	COC(=O)c1ccc(C=C(Cl)c2nc3scc(-c4cccs4)c3c(=O)[nH]2)o1
Z14675562	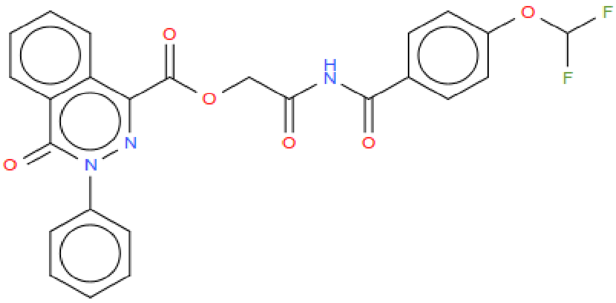	H06	O=C(COC(=O)c1nn(-c2ccccc2)c(=O)c2ccccc12)NC(=O)c1ccc(OC(F)F)cc1
Z111874686	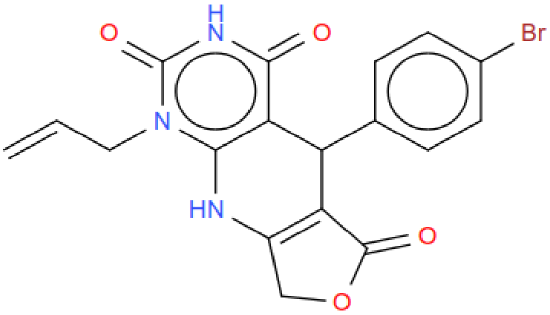	H07	C=CCn1c2c(c(=O)[nH]c1=O)C(c1ccc(Br)cc1)C1=C(COC1=O)N2

Suppl. Table 1. Compounds evaluated in the screen (part 18)

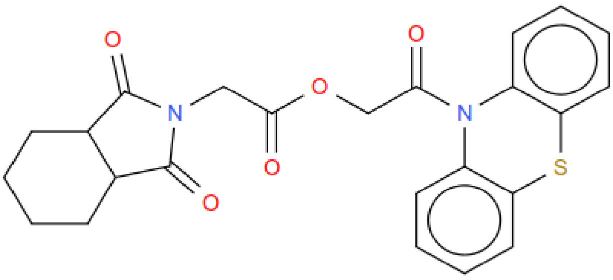

Z21392480 H08 O=C(CN1C(=O)C2CCCCC2C1=O)OCC(=O)N1c2ccccc2Sc2ccccc21

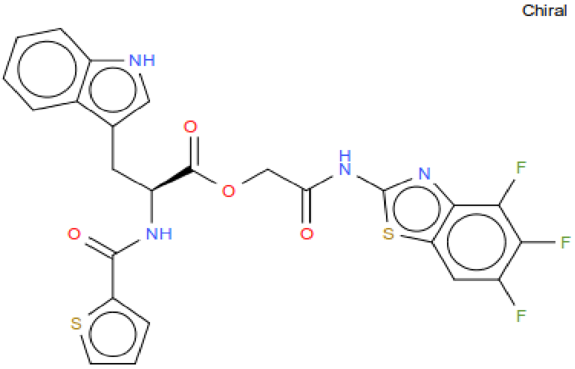

Z56965381 H09 O=C(COC(=O)[C@H](Cc1c[nH]c2ccccc12)NC(=O)c1cccs1)Nc1nc2c(F)c(F)c(F)cc2s1

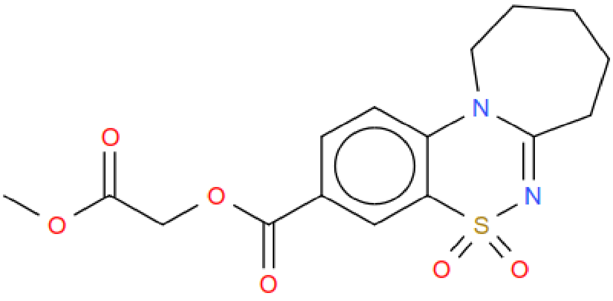

Z54459148 H10 COC(=O)COC(=O)c1ccc2c(c1)S(=O)(=O)N=C1CCCCCN12

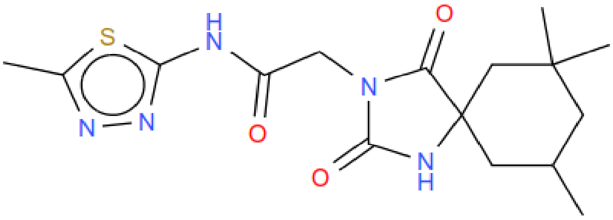

Z29672054 H11 Cc1nnc(NC(=O)CN2C(=O)NC3(CC(C)CC(C)(C)C3)C2=O)s1

Suppl. Table 1. Compounds evaluated in the screen (part 19)

Compound	Adjusted P value	Category
B2	<0.0001	activation
H9	<0.0001	activation
C5	0.0001	activation
E6	0.0008	activation
F1	0.0019	activation
F9	0.1832	non significant
E1	0.1861	non significant
E10	0.3838	non significant
D12	0.6015	non significant
C7	0.8264	non significant
E7	0.9836	non significant
B12	0.9981	non significant
H2	0.999	non significant
B5	0.999	non significant
E9	0.9992	non significant
A4	0.9993	non significant
D2	0.9993	non significant
C4	0.9994	non significant
B4	0.9996	non significant
D4	0.9996	non significant
G11	0.9997	non significant
A11	0.9997	non significant
E12	0.9997	non significant
A3	0.9998	non significant
H6	0.9999	non significant
H12	0.9998	non significant
D7	0.9997	non significant
B9	0.9997	non significant
F10	0.9997	non significant
A8	0.9997	non significant
A12	0.9996	non significant
G8	0.9994	non significant
G10	0.9995	non significant
A7	0.9995	non significant
E11	0.9991	non significant
F5	0.999	non significant
F3	0.9985	non significant
G5	0.999	non significant
E4	0.9989	non significant
B1	0.9988	non significant
D10	0.9988	non significant
D8	0.9916	non significant
F8	0.9987	non significant
G12	0.9987	non significant
A2	0.9986	non significant
B8	0.9986	non significant
D11	0.9986	non significant
H10	0.9985	non significant
F11	0.9984	non significant
F4	0.9984	non significant
C9	0.9984	non significant
H8	0.9983	non significant
G4	0.998	non significant
D9	0.998	non significant
H7	0.9912	non significant
D3	0.7997	non significant
E2	0.9908	non significant
H5	0.9766	non significant
C11	0.9763	non significant
B3	0.9661	non significant
G3	0.5137	non significant
A6	0.9619	non significant
A9	0.954	non significant
E8	0.4234	non significant
A5	0.934	non significant
D1	0.932	non significant
C12	0.9258	non significant
F7	0.9191	non significant
C8	0.893	non significant
H4	0.8456	non significant
G7	0.8173	non significant
H11	0.0643	non significant
F12	0.5664	non significant
B7	0.0816	non significant
E3	0.562	non significant
F2	0.0764	non significant
C3	0.0626	non significant
H1	0.3946	non significant
G2	0.0392	non significant
G1	0.0334	non significant
C10	0.0263	non significant
B11	0.0132	non significant
D6	0.0097	inhibition
G9	0.0073	inhibition
E5	0.0059	inhibition
B6	0.0058	inhibition
C6	0.0057	inhibition
H3	0.0053	inhibition
C2	0.0051	inhibition
D5	0.0046	inhibition
A1	0.0045	inhibition

Suppl. Table 2. Significance levels and p-values for the tested compounds.
